# Functional predictability of universal gene circuits in diverse microbial hosts

**DOI:** 10.1002/qub2.41

**Published:** 2024-04-14

**Authors:** Chenrui Qin, Tong Xu, Xuejin Zhao, Yeqing Zong, Haoqian M. Zhang, Chunbo Lou, Qi Ouyang, Long Qian

**Affiliations:** ^1^ Peking‐Tsinghua Joint Center for Life Sciences Peking University Beijing China; ^2^ CAS Key Laboratory of Microbial Physiological and Metabolic Engineering Institute of Microbiology Chinese Academy of Sciences Beijing China; ^3^ Bluepha Co., Ltd Beijing China; ^4^ Center for Cell and Gene Circuit Design CAS Key Laboratory of Quantitative Engineering Biology Guangdong Provincial Key Laboratory of Synthetic Genomics Shenzhen Key Laboratory of Synthetic Genomics Shenzhen Institute of Synthetic Biology Shenzhen Institutes of Advanced Technology Chinese Academy of Sciences Shenzhen China; ^5^ Center for Quantitative Biology Academy for Advanced Interdisciplinary Studies Peking University Beijing China

**Keywords:** circuit predictability, host‐independent genetic circuits, host‐nonspecific parameters, parts characterization, transcriptional regulatory modules

## Abstract

Although the principles of synthetic biology were initially established in model bacteria, microbial producers, extremophiles and gut microbes have now emerged as valuable prokaryotic chassis for biological engineering. Extending the host range in which designed circuits can function reliably and predictably presents a major challenge for the concept of synthetic biology to materialize. In this work, we systematically characterized the cross‐species universality of two transcriptional regulatory modules—the T7 RNA polymerase activator module and the repressors module—in three non‐model microbes. We found striking linear relationships in circuit activities among different organisms for both modules. Parametrized model fitting revealed host non‐specific parameters defining the universality of both modules. Lastly, a genetic NOT gate and a band‐pass filter circuit were constructed from these modules and tested in non‐model organisms. Combined models employing host non‐specific parameters were successful in quantitatively predicting circuit behaviors, underscoring the potential of universal biological parts and predictive modeling in synthetic bioengineering.

## 1 INTRODUCTION

Model organisms such as *Escherichia coli* have played a pivotal role in the advancement of microbiology and synthetic biology. However, real‐world applications of synthetic biology must engage host cells that accommodate synthetic circuits in specific environments, which renders model organisms inadequate [1, 2]. Over the years, non‐model organisms have shown great potential for industrial and healthcare applications [3] in particular for their unique physiology or morphology under non‐laboratory conditions such as resource limitation and extreme pH or osmotic pressures. As of 2019, roughly 10 microbes have been domesticated for industrial use [4].

Libraries of well‐characterized regulatory components (“parts”) are essential for bottom‐up synthetic biology design [5]. For instance, it is necessary to develop extensive collections of promoters, 5′UTRs, and terminators for the programming and fine‐tuning of basic transcriptional regulatory modules. While widely available for model bacteria like *E. coli*, such collections are lacking for industrial chassis organisms. The intransigence of inherent genetic programs in natural microbes has forced the fragmentation of synthetic biology research into host‐specific domains [6]. For example, Elmore developed a set of synthetic promoters with a wide dynamic range for *Pseudomonas* *putida* for use in bioremediation [7]. Yet, such part collections are accumulating at limiting speeds due to their long development cycles.

To streamline synthetic biological engineering through standardized pipelines, one possible solution is to develop universal biological parts that can function in an extended range of chassis organisms in a plug‐and‐play manner. Intense research efforts have been devoted to the development of universal parts, many of which are of viral origins or are genes engaged in cross‐species interactions and are engineered for enhanced performance and programmability. Examples include recombinases [8], CRISPR/dCas9‐derived systems [9], zinc finger proteins, transcriptional activator‐like effectors, the T7 expression system, and the TetR family of transcriptional repressors [10, 11]. Among these, the T7 system is a well‐established transcriptional module. It consists of the T7 RNA polymerase (RNAP) and its cognate promoter sequences derived from bacteriophages. The system is independent of microbial gene transcription and works effectively in prokaryotic and some eukaryotic organisms [12]. In 2018, Li et al. established a compact T7 expression system on 1 single plasmid for its convenient transfer into 5 different Gram‐negative bacterial strains and 1 Gram‐positive bacterium [13]. A repressor set was then collated, including a set of TetR family repressors (PhIF and LmrA) [11, 14] and bacteriophage repressors (CI434 P22C2 and HKCI) [15, 16]. These repressors have been used broadly in microbial circuit engineering. They can typically recognize specific DNA sequences known as operator sites and inhibit transcription initiation by physically obstructing the binding of the RNA polymerase [17, 18].

In efforts to elevate the functional sophistication of synthetic gene circuits, their rational assembly has become much more reliant on in silico design‐and‐test cycles, which necessitates model building with detailed quantification of the underlying parts [19]. For circuits built with universal parts in particular, model predictability relies critically on how much the synthetic program can be decoupled from specific cellular physiology, or put another way, can we at some level of abstraction quantify the working environment a host “operating system” [20] provides to a synthetic program. On the cellular level, a recent model identified specific parameters at the interface of heterologous gene circuits and host homeostasis [1]. On the circuit level, despite ongoing efforts in parameterizing synthetic circuits via biophysical, dynamical, and Boolean modeling in a focal species, demonstrating fair predictive powers [1, 21, 22], the effects of parameter variation on circuit behavior in non‐model organisms have not been interrogated in depth. In principle, one can divide the parameter space of a synthetic circuit into host context‐dependent and ‐independent sectors. Intrinsic biochemical parameters like Hill coefficients are likely invariant, while a large number of reaction rates such as those of basal transcription and translation are likely sensitive to the host’s genetic background and physiological state [23]. However, the majority of universal parts have not been characterized in sufficient detail to enable predictive modeling to approach the precision to be practically applied in a different species or even in sister strains of the same species [24].

In this study, we use the T7 activator module and the different transcriptional repressor module as a model system to investigate the parameter universality across four different host microbes: the filamentous bacterium *Streptomyces albus*, the Gram‐positive *Corynebacterium glutamicum*, and the Gram‐negative *Pseudomonas entomophila* along with the model organism *E. coli.* By building genetic circuits implementing the NOT gate and a non‐monotonic response function [25] from universal parts, we report the effects of host‐specific contexts on synthetic circuit performance in the form of parametrized dose‐response curves. Sensitive and insensitive parameters are identified and quantified, which concretely characterize the cross‐species universality of the class of host‐independent universal parts.

## 2 RESULTS

### 2.1 Overview of research

**FIGURE 1:**
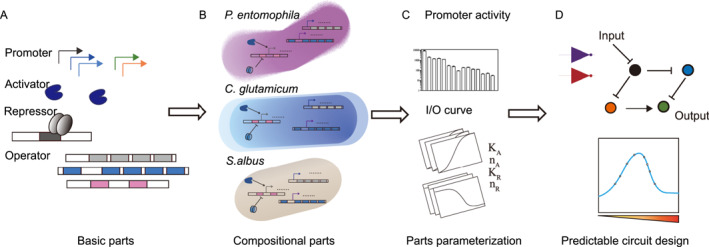
The bottom‐up design principle of genetic circuit across diverse species. (A) Parts derived from bacteria and phages are subjected to standardization. (B) Reliable physical and functional composition of standard biological parts are constructed across diverse organisms. (C) Combinational parts are characterized and parameterized via modeling. (D) Genetic circuits are designed based on models and simulations to produce predictable responses across various species.

We developed a quantitative framework to explore the universality of biological parts and their reliability in non‐model organisms. Two universal transcriptional regulatory toolkits were interrogated in our pipeline. One was an activator library composed of T7 RNAP and its cognate promoters. The other is a repressor library [14] and their operator sequences (Figure 1A). First, we quantified respectively the activities of the RNAP, the transcription repressors, and their corresponding cis‐acting sequences in four different species (Figure 1B). Next, we constructed inducible transcriptional regulatory modules with either toolkit and measured the steady‐state circuit output via flow cytometry. By fitting experimental data to equilibrium models of transcription activation or repression, we evaluated the robustness of key parameters involved in both modules against host cellular contexts (Figure 1C). Finally, host‐specific and host‐nonspecific parameters obtained above were employed in combined models of circuits involving both toolkits in *C*. *glutamicum*, to test the predictability of circuit behavior from underlying universal parts with detailed quantitative characterizations (Figure 1D).

### 2.2 Multi‐host compatibility and predictability of T7 promoters

Synthetic transcriptional regulation often involves information integration at the promoter region, where the core promoter sequence interacts with the RNA polymerase, which is further affected by transcriptional regulators that dock on nearby cognate operator sites [26]. The predictability of downstream gene expression output crucially relies on modularized promoter organization, that is, element‐wise insulation, and quantitative activity profiles of each sequence element. A previous study has shown the insulated structure of composite promoters consisting of various T7 core promoters in combination with different repressors’ operators [15]. Most genes change expression levels across different environmental conditions and genetic contexts [27, 28]. The activities of synthetic T7 core promoters have been characterized quantitatively in a single host under different cultural conditions [29, 30]. However, their reproducibility in different organisms has not been evaluated.

**FIGURE 2:**
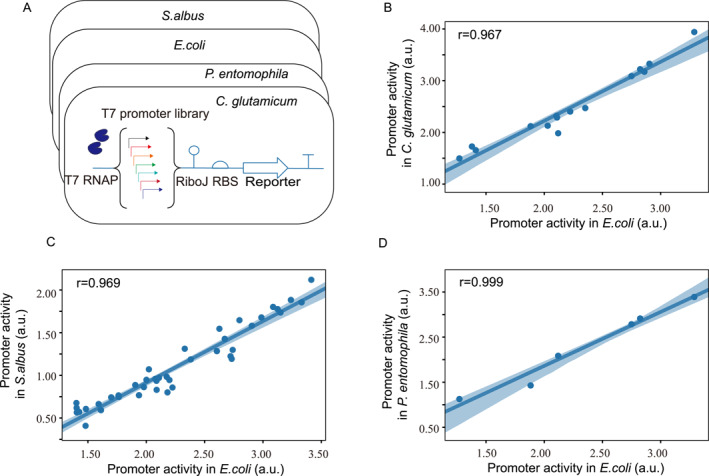
The relative activities of promoters are preserved among diverse species. (A) Diagram depicting the assessment of promoter activity. Each promoter was placed upstream of the same reporter protein in compatible plasmids and assessed for its expression in a range of host organisms. The four master strains also incorporate T7 RNAP, driven by the host‐specific promoters. Promoter activities were assessed using superfolder GFP as the reporter and quantified as the arithmetic mean of flow cytometry fluorescence data. The T7 promoter library consists of 15 sequences individually tested in *Corynebacterium glutamicum*, 6 sequences in *Pseudomonas entomophila*, and 42 sequences in *Streptomyces*, respectively. (B) Correlation between promoter activities in *Escherichia coli* (on the *x*‐axis) and *C. glutamicum* (on the *y*‐axis). (C) Correlation between promoter activities in *E. coli* (plotted on the *x*‐axis) and *Streptomyces albus* (plotted on the *y*‐axis). (D) Correlation between promoter activities in *E. coli* (*x*‐axis) and *P. entomphila* (*y*‐axis). In (B–D), the 95% confidence band was visualized as the blue shaded area.

We sought to investigate the consistency of activities for synthetic T7 promoters across a panel of organisms. To obtain accurate measurements of promoter activities, a simple and effective experimental system was developed: on one plasmid, a host‐specific constitutive promoter was used to drive the expression of T7 RNAP; on a different plasmid, one of the T7 promoter variants was placed upstream of a *sfgfp* gene which served as the transcriptional readout, followed by a T7‐efficient transcriptional terminator. In addition, a RiboJ insulator [31] was introduced to eliminate interference between promoters and translation signals to improve the modularity of elements (Figure 2A).

The panel of synthetic T7 promoters tested in this study were of 17 base pairs (bp) in length, and drove gene expression by up to 180‐fold activation in *E. coli* (Supplementary Figure S1, Supplementary Table S1). Here, their promoter activities were independently evaluated in *E. coli*, *C. glutamicum*, *P. entomophila* (Figure 2A). By measuring cellular fluorescence through flow cytometry, the steady state *sfgfp* expression levels were determined for each host species with each promoter construct. In a study by Zhao et al. [32], data regarding the promoter activities in *S. albus* were also reported. In this paper, we leverage the dataset to explore whether T7 promoters maintain their relative activities across different hosts. Pairwise comparison of promoter activities between the reference species *E. coli* and a non‐model chassis organism on the log‐log scale revealed near‐linear relationships for all T7 promoter variants, yielding *R* ^2^ values of 0.95, 0.93, and 0.97 by parametric linear regression and Pearson correlations coefficients of 0.967, 0.969 and 0.999 between promoter activities in *E. coli* versus those in *C. glutamicum*, *S. albus* and *P. entomophila*, respectively (Figure 2B–D, Supplementary Figure S1A,B). These results indicate the quantitative cross‐species universality of the T7 promoter library. The linear scaling as we obtained here enables the extrapolation of promoter activities when a new T7 promoter variant is employed in a non‐model organism that has already been tested for a couple of other T7 promoter variants.

The fact that the T7 expression system is entirely non‐existent in microbial genomes [33] and that the promoter activities strongly depend on binding affinities between the promoter sequence and the T7 RNAP contribute to the superior linear consistency of the T7 system as opposed to other species‐specific expression systems. However, we emphasize that the linear scaling is concentration‐dependent and it may not hold when the viral polymerase is overexpressed. A saturation effect may occur when promoters become almost fully occupied by high copy numbers of T7 RNAP, in which case the transcriptional output levels off, leading to the breakdown of cross‐species linearity (Supplementary Figure S2A,B). This situation may be rescued by down‐regulating the expression level of T7 RNAP. We created an RBS library to tune the constitutive expression levels of T7 RNAP in different species. The screen identified medium‐to‐high strength RBSs that exhibited linear scaling across species for all promoter variants (Supplementary Figure S2C).

### 2.3 Model parametrization reveals host‐independent properties for the T7 activation module

**FIGURE 3:**
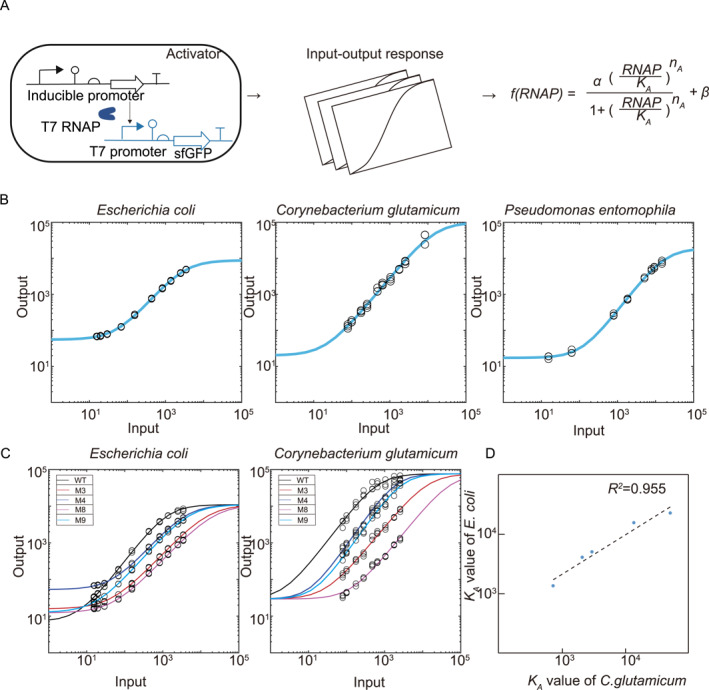
Parameterization of the T7 transcriptional activator. (A) Scheme of genetic circuits for the characterization of the T7 transcriptional activator. The input RNAP was quantified by sfGFP fluorescence which was transcribed from a separate cassette under the control of the same input promoter. Each circuit was implemented in a distinct species and parameter fitting was performed using the biophysical model to characterize the interactions between the promoter and the transcriptional activators. (B) Experimental measurements and parameter fitting of response functions for T7 RNAP were conducted in *Escherichia coli*, *Corynebacterium glutamicum*, and *Pseudomonas entomophila*. Data represent the means ± SD from at least three replicate experiments. (C) Global fitting curves were generated to parameterize various core promoters in *E. coli* and *C. glutamicum*. (D) The correlation between *K*_*A*_ values in *E. coli* and *C. glutamicum*.

The linear scaling of T7‐based gene activation suggested that certain generic biophysical parameters of the genetic module governed its activities across organisms. Such parameters are ideal candidates for biological part standardization [5] and are extremely useful for model‐directed design, in particular, the choice of biological parts that reliably generate desired outputs in non‐model organisms. Therefore, we quantitatively studied transcription activation mediated by the interaction between core promoter sequences and T7 RNAP, which together comprised the activator module. A robust and well‐established way to characterize modules is in terms of their input‐output relationships (i.e.,dose‐response curves). An experimental framework reported in a previous study [15] was employed. To measure circuit outputs, the transcriptional activator T7 RNAP was driven by an inducible promoter (Ptac or Plux, depending on the organism) and activated the expression of a downstream *sfgfp* reporter gene via different T7 promoter sequences. For circuit inputs, in separate experiments, the *sfgfp* gene was placed under the same inducible promoter to reflect the expression levels of T7 RNAP at specific inducer concentrations. As T7 RNAP expression was tuned in a range spanning three orders of magnitudes, the output fluorescence was taken at steady states (Figure 3A).

The experimental input‐output curves were fitted to a biophysically parametrized Hill function where the circuit output, *f*(*RNAP*), can be written in a dimensionless form [15]:

(1) $$f\left(\mathrm{RNAP}\right)=\frac{\alpha {\left(\mathrm{RNAP}/K_{A}\right)}^{n_{A}}}{1+{\left(\mathrm{RNAP}/K_{A}\right)}^{n_{A}}}+\beta$$

where *RNAP* represents the expression level of the activator, for which we took the input fluorescence as a proxy, *α* is the maximal activity of the promoter, *n*_*A*_ indicates the effective cooperativity of activation, *K*_*A*_ is the concentration of the activator yielding half‐maximal expression, and *β* quantifies the basal expression not driven by T7 RNAP.

Model fitting to experiments of T7 and a selected promoter (M4 for *E. coli*, M3 for *C. glutamicum*, and M5 for *P. entomophila*) showed consistently weak cooperativity for T7 RNAP across all three species, with the fitted *n*_*A*_ being 1.26, 1.28, 1.38 in *E. coli*, *C. glutamicum* and *P. entomophila*, respectively (Figure 3B, Supplemental Table S2). The cooperativity fitted in *E. coli* was consistent with that measured by Zong et al [15]. More importantly, there was no significant difference between *n*_*A*_ values in the three species, suggesting that the Hill coefficient for T7 RNAP is host context‐independent and thereby defines the universality of T7 RNAP. The Hill constant, *K*_*A*_, reflects the effective binding affinity or the dissociation constant between the promoter sequence and T7 RNAP. Next, we measured dose‐response curves of different T7 promoter variants in *E. coli and C. glutamicum* (Figure 3C), from which the *K*_*A*_ values could be determined. The dose‐response curves shifted toward higher T7 RNAP concentrations when *K*_*A*_ increased (Figure 3C). However, *K*_*A*_ for different promoter sequences remained linearly scaled between *E. coli* and *C. glutamicum* (*R* ^2^ = 0.955) (Figure 3D). This suggested that the linear regime for promoter universality, as observed in Section 1, was wide enough for tunable activation as is often needed in synthetic circuits. These globally fitted parameters of the activator module were fed into our characterizations of a NOT gate genetic system in the next set of experiments (Supplementary Table S2).

### 2.4 Host‐independent properties of the different repressor module with variable promoter architectures

Transcription factors can act as repressors that reduce the transcription rate when bound to operator sites proximal to the core promoter of the regulated gene. Both activators and repressors form the essential repertoire enabling complex transcriptional regulation. We next tested the universality of the repressor modules composed of the transcriptional repressors and their cognate operator sequences in different organisms.

**FIGURE 4:**
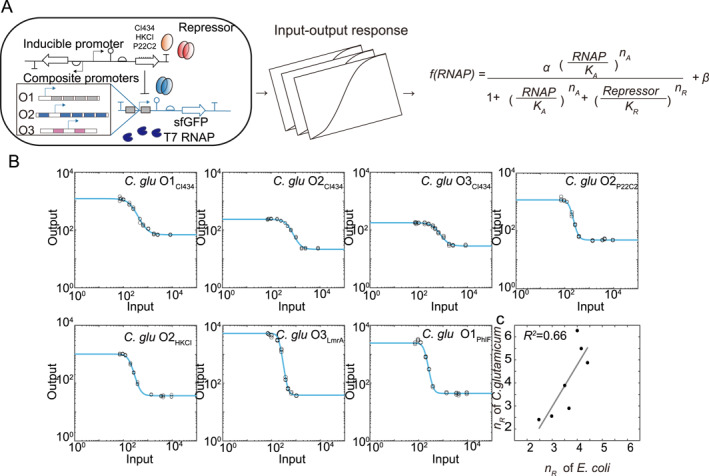
Parameterization of transcriptional repressors. (A) Diagram depicting genetic circuits used to evaluate transcriptional repressors. The transcriptional activator was expressed constitutively. (B) All repressor‐promoter pairs were converted to NOT gates and characterized. (C) The correlation of *n*_*R*_ value between *Escherichia coli* and *Corynebacterium glutamicum*.

A series of NOT gates were constructed with constitutively expressed T7 RNAP, inducible repressors (by IPTG‐Ptac or AHL‐Plux, depending on the organism) and composite promoters. Five repressors were tested in both *E. coli* (serving as a reference organism) and *C. glutamicum* to probe the genetic context effect on the repressor modules. For each repressor and its cognate operator sequence, three composite promoter architectures, termed O1, O2, and O3, were employed with one or zero operators upstream of the M4 variant of the T7 promoter and another one or four operators downstream of it (Figure 4A). The M4 promoter was chosen for its relatively high activation strength and its large regulatory range. Variable numbers of consecutive operator repeats in these architectures enabled the repressors’ effective binding cooperativities to diversify [15]. Insulated transcriptional elements enable precise design, as shown below. Differentially subdued expression of the reporter *sfgfp* gene was obtained for these NOT gates (Figure 4A, Supplementary Figure S3). Again, the input repressor concentration was measured separately in simple constructs where *sfgfp* was driven by the Ptac or the Plux promoters.

We fitted the dose‐response curves of the NOT gates to a biophysically parametrized Hill function:

(2) $$f\left(\mathrm{RNAP},\text{Repressor}\right)=\frac{\alpha {\left(\mathrm{RNAP}/K_{A}\right)}^{n_{A}}}{1+{\left(\mathrm{RNAP}/K_{A}\right)}^{n_{A}}+{\left(\text{Repressor}/K_{R}\right)}^{n_{R}}}+\beta$$

where Repressor represents the repressor concentration as measured by the input fluorescence, *n*_*R*_ indicates the effective cooperativity in repression, *K*_*R*_ is the concentration of the repressor achieving $\frac{100}{2+{\left(\frac{\mathrm{RNAP}}{K_{A}}\right)}^{n_{A}}}\%$ repression, and all activation‐related parameters were obtained from fitted results for the M4 core promoter.

A comparison of fitted *n*_*R*_ between *E. coli* and *C. glutamicum* for all tested repressor‐composite promoter pairs was shown in Figure 4C. Decent linearity was observed (*R* ^2^ = 0.66, *r* = 0.824), and *n*_*R*_ was much less affected by the host genetic context than by the repressor module’s genetic nature, that is, the promoter architecture and the identity of the repressor. The Hill coefficient of regulatory modules is critical for many circuit behaviors. For example, the essential condition for a bistable toggle circuit is that at least one of the repressors has a Hill coefficient *n*_*R*_ > 1 [34]. The Hill coefficient was previously maneuvered by engineering protein oligomerization domains or collocating multiple synthetic operators [35]. The linear scaling of the Hill coefficient across species supports the view that the cooperativity of a repressor module is insensitive to cellular contexts, which serves a premise for the rational predesign of genetic circuits in non‐model organisms.

We emphasize that for the linear scaling to hold, the expression level of the repressor should be within an appropriate range, as was the case for T7 RNAP in Section 1. We tuned the expression level of repressors by changing the strength of their RBSs guided by the RBS calculator [36] to make sure that the NOT gates worked in the linearity regime. The *K*_*R*_ value, also known as the repressor dissociation constant, can vary between different species and be influenced by the host translation machinery. Different RBSs shifted the response threshold, suggesting limited transferability.

For some of the repressor‐composite promoter pairs tested, we noticed that there were unusually high transcriptional activities even in the absence of T7 *RNAP*, suggestive of unintended emergence of promoters that could be recognized by the host cell’s endogenous transcriptional machinery (Supplementary Figure S4). This phenomenon has been reported in a previous study [15]. Promoter finding algorithms can aid in ridding the synthetic sequence of cryptic promoters [37].

### 2.5 Predictive design of a logic gate and a feed‐forward loop in a non‐model organism

While research has shown that behaviors of assembled circuits can be effectively predicted from underlying components that have been well characterized in the same host and controlled laboratory environments [15], the reliability of circuit behavior in non‐model organisms or in uncalibrated environments has not yet been evaluated. Working in *C. glutamicum*, we demonstrated how quantitative characterization of universal parts and predictive modeling work together to guide the predictive design of composite genetic circuits.

**FIGURE 5:**
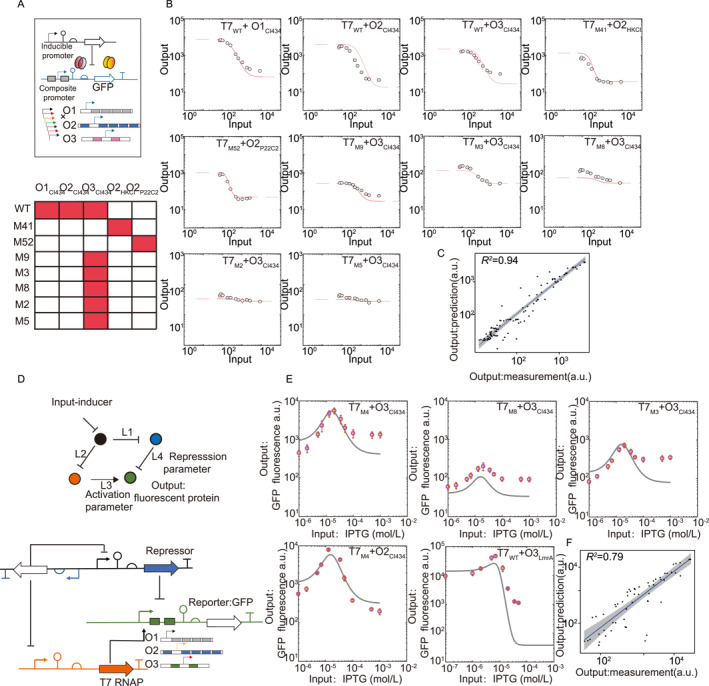
Predictive design of NOT gates and synthetic IFFL networks in *Corynebacterium glutamicum*. (A) A diagram for the construction of the compositional parts. The upper panel displays a set of promoters and synthetic operator combinations for the design of composite promoters functioning as NOT gates. Experimental evaluations were conducted for the combinations highlighted in red in the lower panel. (B) The steady‐state response functions for 10 repressor‐operator pairs. The experimental data is denoted by circles, while fitted results are represented by solid lines. Data represent the means ± SD from at least three replicate experiments. (C) Correlation between experimental measurements and model predictions. (D) The topology of the incoherent feed‐forward (IFF) circuit consists of arrows and blunt‐end arrows representing activation and repression, respectively, and its genetic implementation. (E) The response functions of IFF circuits were both predicted (lines) and experimentally determined (circles). Data represent the means ± SD from at least three replicate experiments. (F) Correlation between experimental measurements and model predictions for all 57 data points.

NOT gates are essential transcription modules that underlie many synthetic circuits. We hypothesized that for the two toolkits we characterized in detail, activation‐derived parameters of different core promoters and repression‐derived parameters of different repressor‐operator pairs could be unified to model composite promoters and multi‐layer circuits with high precision. To test the hypothesis, we genetically constructed 10 combinations of T7 promoter variants and synthetic operator arrays and experimentally measured the dose‐response curves of the resulting NOT‐gates (Figure 5A). Remarkably, with values of host‐specific *α*, *β*, *K*_*R*_, *RNAP* and non‐specific *n*_*A*_, *n*_*R*_, *K*_*A*_ gleaned from previous essays, dose‐response curves of these newly designed NOT gates were reasonably predicted (Figure 5B). The *R* ^2^ value was 0.94 across all measurement points pooled from all NOT gate constructions (*n* = 108) with mean fold errors^1^ of 1.05–1.48 for each individual construct, validating the combined predictive modeling approach for genetic modules constructed from well‐quantified universal toolkits, and the good transferability of the values of the host‐independent parameters between diverse species (Figure 5C).

As an example application, we next showed that a concentration band pass filter [38] function based on the incoherent feed‐forward (IFF) circuit can be predictably designed in *C. glutamicum*. The band pass filter refers to the circuit’s steady state behavior that over a certain range of input doses, the output responds only to the intermediate levels of inputs, whereas the output diminishes at both low and high levels of inputs. Such a function is fundamental for biological processes such as morphogenesis and could be particularly useful for tissue engineering. Besides, experiments and theory have outlined some of the critical dynamic functions that can be carried out by IFF circuits, such as the adaptative response that underlies important sensory functions from bacterial chemotaxis to human vision [25, 39].

Our IFF circuits employed the same architecture as that used in Zong et al. [15] (Figure 5D). The input signal, IPTG, simultaneously controlled the expression of T7 RNAP and a repressor (CI434 or LmrA) through the LacI‐repressed Ptac promoter. The output *sfgfp* gene was regulated antagonistically by T7 RNAP and the repressor through a composite promoter sequence made up of a T7 promoter and the operator array for the repressor. The input signal was transmitted through the two parallel regulatory limbs, whose relative activities were tuned independently by changing the corresponding sequence elements in the composite promoter to generate positive responses only at intermediate IPTG concentrations.

The behavior of the band‐pass filter circuit was modeled by coupling equations of repressive transcriptional regulations by IPTG and Equation (2), with parameters derived from previous sections (Supplementary Tables S3–S5, Supplementary Figure S5). To test the model’s predictability, we assembled five IFF circuits using three T7 promoter variants, two repressors and three operator architectures in combination, and quantified the output response to IPTG input concentrations spanning three orders of magnitudes (Figure 5E). The experimental data correlated very well with model predictions (*R* ^2^ = 0.79), with mean fold error 1.59–1.95 and 3.74 when CI434 or LmrA was used for the repressor module, respectively. Moreover, circuit responses were highly predictable. We found almost perfectly aligned peak heights and response ranges of IPTG concentrations (*R* ^2^ = 0.949 and 0.982, respectively) between experimental results and model predictions (Supplementary Figure S6).

## 3 DISCUSSION

The synthetic biological parts studied in the current research have been shown to function in a wide panel of organisms, and their activities, especially with respect to modularity, have been extensively quantified in the most common experimental microbes. Universal biological parts are expected to function independently of the genetic background they are placed into and be minimally influenced by unpredicted conditions. Our study provided strong evidence for the host transferability of these universal transcriptional parts. Strikingly, we found their activities in non‐model organisms obey linear scaling within a wide range. Further, these standard and insulated parts were reliably reused in combination with one another to reproduce quantitatively consistent transcriptional regulatory functions in a non‐model microorganism with industrial significance. Our results suggested that with universal parts, predictive models learned from a well‐characterized bacterium can be generalized for other potentially divergent microorganisms. It is conceivable that the parameters obtained for universal parts are not exclusively applicable to the few organisms tested in the current study. In particular, it would be interesting to test these universal parts in host cell lines relevant to biomedical applications.

By employing simplified models of transcription regulation, we were able to dissect the key parameters underlying part universality. We found hill coefficients and dissociation constants of each DNA‐binding‐based regulatory module to be robust against the host cell’s genetic context, corroborating the conjecture that intrinsic aspects of universal parts reliably work independently of the host operating system. In fact, these parameters are more often than not the defining features of complex synthetic gene circuits, whose function shows high sensitivities toward these parameters, and are thus frequently the targets for parts engineering [16, 40].

Our simplification of the models for various transcriptional regulations, whether standalone or in combination, was justified. The addition of the second‐order term describing the non‐equilibrium effects, as proposed in Zong et al. [15], did not alter the fitting results significantly. The range within which linear scaling remained valid was adjusted by RBS strengths which affected the equilibrium expression levels of the transcriptional regulators. In our system, basal expression and maximal expression were host‐specific. These parameters become important when multiple synthetic genetic modules are serially connected. It is likely that they can be manipulated by extra feedback regulations or by fine‐tuning host resource allocation [39, 41]. As far as modeling is concerned, these parameters can be included as supra‐parameters or can fit in more complex models coupling local regulatory functions to circuit‐circuit and circuit‐host interactions [42, 43]. In this sense, equilibrium models of individual modules can be joined to generate predictions for layered synthetic circuits. Of course, for other circuit behaviors, such as its dynamics, the set of relevant parameters might expand; and for complex dynamic properties such as those of a bi‐stable switch, stochastic models might be employed to precisely predict circuit behaviors.

It is also important to mention that part parameterization by mathematical modeling is often evaluated at experimental steady states. For example, previous research has revealed the model system *E. coli* has a bi‐plateau mode of gene expression and the dynamically active first plateau is conducive to the predictability of circuit‐level assemblies and captures the intrinsic properties of basic parts [44]. In this study, experimental measurements were made at non‐specific stages of cellular growth, for either the optimal measurement condition had not been characterized such as in *P. putida* or the condition was not met such as in *C. glutamicum*. Under non‐standard measurement conditions, circuit behavior can be influenced by the host’s translational machineries, even though at the transcription level it is autonomous [45]. Conversely, synthetic gene circuits may affect host physiology which then acts back on circuit outputs. However, the T7 system and the repressor system used here did not cause detectable cytotoxicity, and our measurements from non‐standard growth phases indicated the robustness of these universal parts.

The scaling up of synthetic biology crucially depends on our ability to make the design and construction of large genetic circuits more reliable and predictable in mildly modified host cells with applicational significance. Given their broad applicability and quantitative robustness as shown in this work, universal parts may enable rapid prototyping of genetic circuits in novel organisms, and they can be quickly characterized with automatic experimental platforms. In recent years, with bioinformatics mining, directed evolution, and rational engineering [6, 46, 47, 48], the plethora of natural gene offerings are being leveraged for their unique properties to apply in synthetic biological engineering. We expect universal parts, including transcriptional units, proteases, nucleases and many more, to accumulate over time. Systematic evaluations of their universalities, as aided by parameterized modeling, should be applied to demarcate the boundary of predictive synthetic biological design, especially in mammalian cells [49].

## 4 MATERIALS AND METHODS

### 4.1 Strains and cell culture conditions

*E. coli* DH10B, *C. glutamicum* ATCC 13032 (wild type), *Pseudomonas* LAC31, and *S. albus* J1074 were used as the host organisms. The culture temperatures were 37°C and 30°C. *E. coli* was cultivated in Luria−Bertani (LB) medium or M9 medium supplemented with ampicillin (50 μg/mL) and chloramphenicol (23 μg/mL). *C. glutamicum* was cultivated in LB medium supplemented with kanamycin (20 μg/mL) and chloramphenicol (7.5 μg/mL). *Pseudomonas* was cultivated in LB medium supplemented with kanamycin (10 μg/mL) and chloramphenicol (170 μg/mL). *S. albus* strains were cultured on the MS medium for sporulation and conjugation supplemented with apramycin (50 μg/mL).

### 4.2 Plasmid construction

All cloning was performed in *E. coli* (DH5α and TOP10 strain for cloning) and S17‐1(conjugative donor strain), and constructs were confirmed by sequencing before retransformation into the relevant species. The final vector is sequenced to ensure fidelity and then reused as a modular part for constructing other genetic circuits.

A one‐pot Golden Gate assembly reaction: 0.4–1 μL of T4 Ligase (400,000 U/mL) + 2 μL of T4 Ligase buffer (10×), 40 fmol for all vectors used in the reaction, ddH_2_O up to a final total volume of 20 μL. The thermocycler program used for all assemblies included: 50 cycles of [5 min at 37°C followed by 10 min at 16°C]; 1 step of 15 min at 37°C, 1 step of 5 min at 50°C and 1 final step of 5 min at 80°C. Two basic plasmids for *E. coli*: pRT and pRG; two basic plasmids for *C. glutamicum*: pEC‐XK99e and pXMJ19; two basic plasmids for *P. entomophila*: pSEVA321 and pSEVA341; two basic plasmids for *S. albus*: pTHS‐XGSN* and pPAS‐PT.

### 4.3 Standard flow‐cytometer measurements

Each reporter strain was inoculated in three technical replicates per experiment. All data were recorded with appropriate voltage and contained at least 10,000 cells.

*E. coli*: Plates were incubated for 12 h at 37°C in a Digital Thermostatic Shaker (AOSHENG) at 1000 rpm. The precultures were diluted 196‐fold with the M9 medium. After 3 h of growth, the cultures were further diluted 700‐fold with M9 medium (containing inducer) and incubated for another 6 h.

*C. glutamicum*: Plates were incubated for 24 h at 30°C in a shaker at 1000 rpm. The precultures were diluted 196‐fold with the LB medium. After 8–10 h of growth, the cultures were further diluted 200‐fold (containing inducer) with LB medium and incubated for another 29 h.

*P. entomophila*: Plates were incubated for 24 h at 30°C in a shaker at 1000 rpm. The precultures were diluted 500‐fold with LB medium (containing inducer) and incubated for another 18 h.

*S. albus*: Spores of *S. albus* were used to inoculate a 24‐well plates containing 2‐mL TSB medium for 48 h at 28°C in a shaker at 500 rpm. The precultures were diluted 100‐fold with TSB medium and incubated for another 48 h.

Finally, samples were treated with kanamycin at a final concentration of 2 mg/mL to inhibit protein synthesis and analyzed using LSRII flow cytometer (BD Biosciences) with appropriate voltage settings. EGFP fluorescence values were obtained from a minimum of 50,000 cells in each sample. The geometric mean of the EGFP fluorescence per cell was calculated using Flowing Software version 2.5.1.

### 4.4 Media and buffers

Bacterial strains were grown in LB (10 g/L tryptone, 5 g/L yeast extract, and 10 g/L NaCl) (Fisher Scientific) liquid media or on agar plates (media with 1.5% agar powder) for plasmid construction and strain maintenance. M9 media for testing (5× M9 salts, 1 mM thiamine hydrochloride, 0.4% glucose, 0.2% BD casamino acids, 2 mM MgSO_4_, and 0.1 mM CaCl_2_). MS medium used for the routine cultivation of *Streptomyces* (20 g/L soybean flour, 20 g/L mannitol, and 20 g/L agar). Inducer: IPTG (isopropyl–d‐1‐thiogalactopyranoside) or AHL (N‐(3‐Oxohexanoyl)‐l‐homoserine lactone). Phosphate‐buffered saline(PBS): 8 g/L NaCl, 0.2 g/L KCl, 1.44 g/L Na_2_HPO_4_, 0.24 g/L KH_2_PO_4_. For all Golden Gate assembly reactions, we used: 0.4 µL of TypeIIS enzyme (either BsaI from NEB). Antibiotics: ampicillin; chloramphenicol; kanamycin. Unless otherwise specified, reagents were purchased from Sigma‐Aldrich.

### 4.5 Parameter fitting

The data produced and obtained from these characterization circuits needs to be of sufficient quality such that predictions can be made for a novel compositional circuit. The best‐fit value of parameters describing the dynamic features of promoter cores and operators were obtained by fitting using the “fit” function in MATLAB (version 2020a). The 95% confidence interval was computed using “Bootstrap sampling”. The predictions for the combinations of promoter cores and operators (NOT gate) were performed using the mathematical function. The parameter values were retrieved from the fitting results of the characterization data. The steady‐state solutions for transcriptional IFFL networks were predictably calculated using MATLAB (version 2020a).

## AUTHOR CONTRIBUTIONS

Long Qian, Qi Ouyang and Chunbo Lou conceived and designed the study; Tong Xu, Chenrui Qin, Xuejin Zhao, Haoqian M. Zhang, and Yeqing Zong performed in vivo experiments; Chenrui Qin analyzed the experimental data; Long Qian and Chenrui Qin wrote the manuscript; Long Qian edited the manuscript.

## CONFLICT OF INTEREST STATEMENT

The authors Chenrui Qin, Tong Xu, Xuejin Zhao, Yeqing Zong, Haoqian M. Zhang, Chunbo Lou, Qi Ouyang and Long Qian declare that they have no conflict of interest or financial conflicts to disclose.

## ETHICS STATEMENT

This article does not contain any studies with human or animal subjects performed by any of the authors.

## Supporting information

Supporting Information S1

## ENDNOTE

^1^
Mean fold error = 10^(sum(abs(log10(dy) – log10(pred_out)))/12).
