# Supplementary material for: Functional predictability of universal gene circuits in diverse microbial hosts
Source: Quant Biol. 2024 Apr 14;12(2):129–40. doi: 10.1002/qub2.41 (PMC12806508; doi:10.1002/qub2.41)
Supplement: Supplementary file 1 — Supporting Information S1 [file QUB2-12-129-s001.docx]

**Supporting Information**


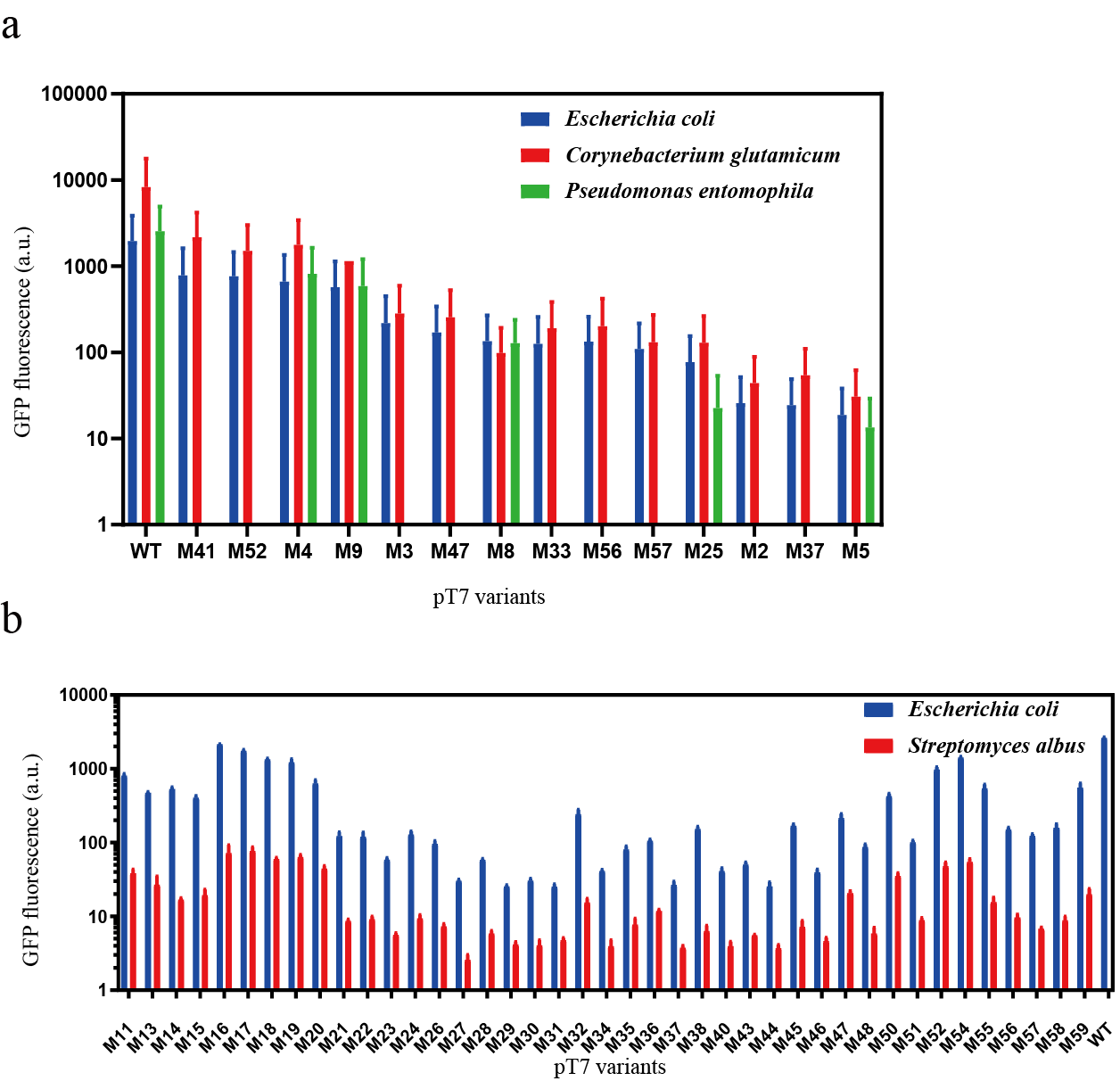
**Supplementary Figure 1. The activity of T7 promoters among different species.** The experimentally measured activities of various T7 core promoters in *E. coli*, *C. glutamicum*, *P. entomphila*, and *S. albus* are shown. The promoter activity was measured using super folder GFP as the reporter and quantified as the arithmetic mean of flow cytometry fluorescence data. Error bars show the standard deviation of three independent replicates.


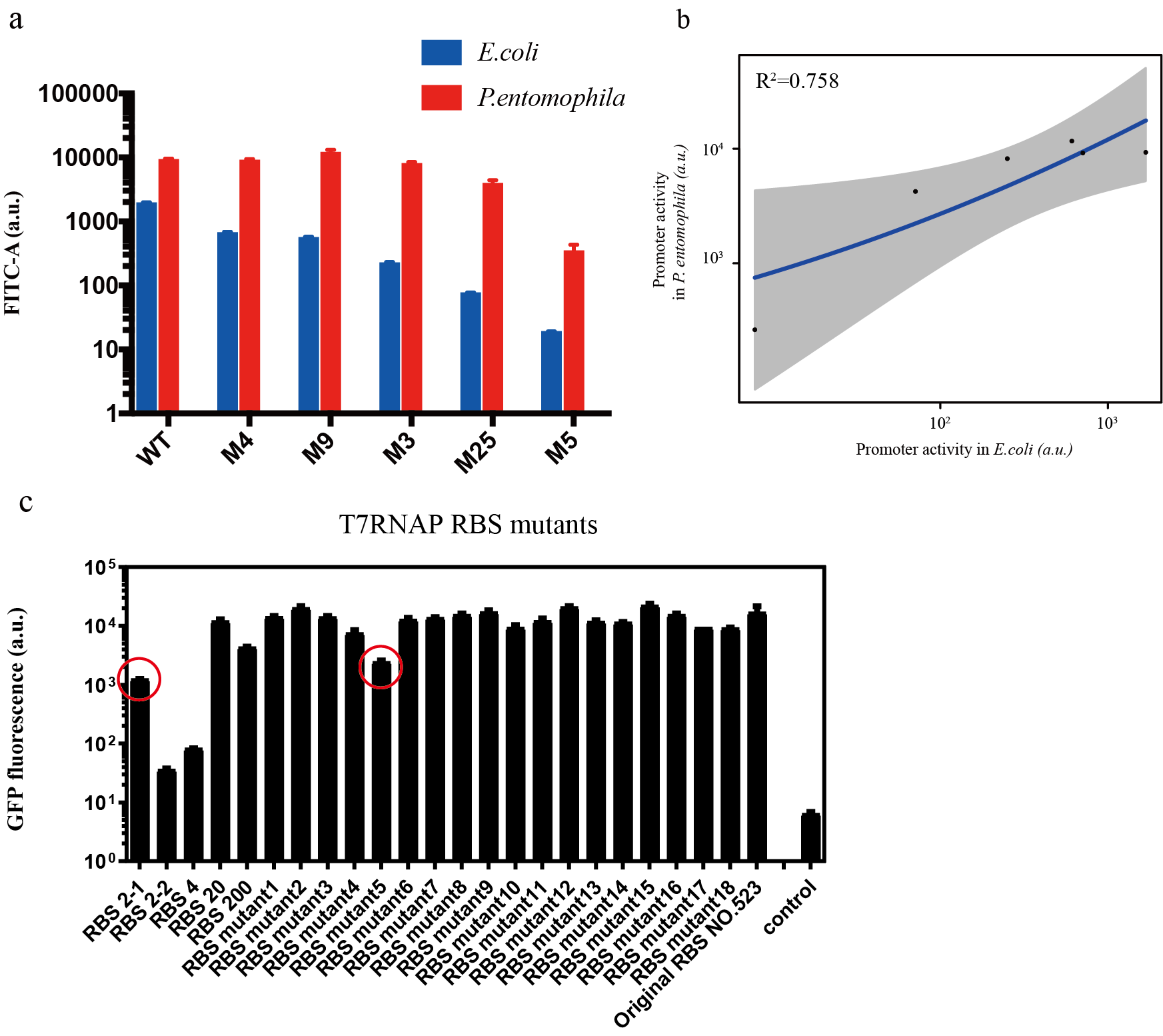
**Supplementary Figure 2. Saturation of promoter activity.** (a) The promoter activity measured in *E. coli* and *P. entomphila* when the T7 RNAP was overexpressed. (b) The correlation between promoter activity in *E. coli* (x-axis) and *P. entomphila* (y-axis) when the T7 RNAP was overexpressed. ﻿R^2^ was calculated by linear regression. (c) the activity of the T7 promoter when Different RBS sequences were combined with the CDS of the RNAP. Error bars show the standard deviation of three independent replicates.


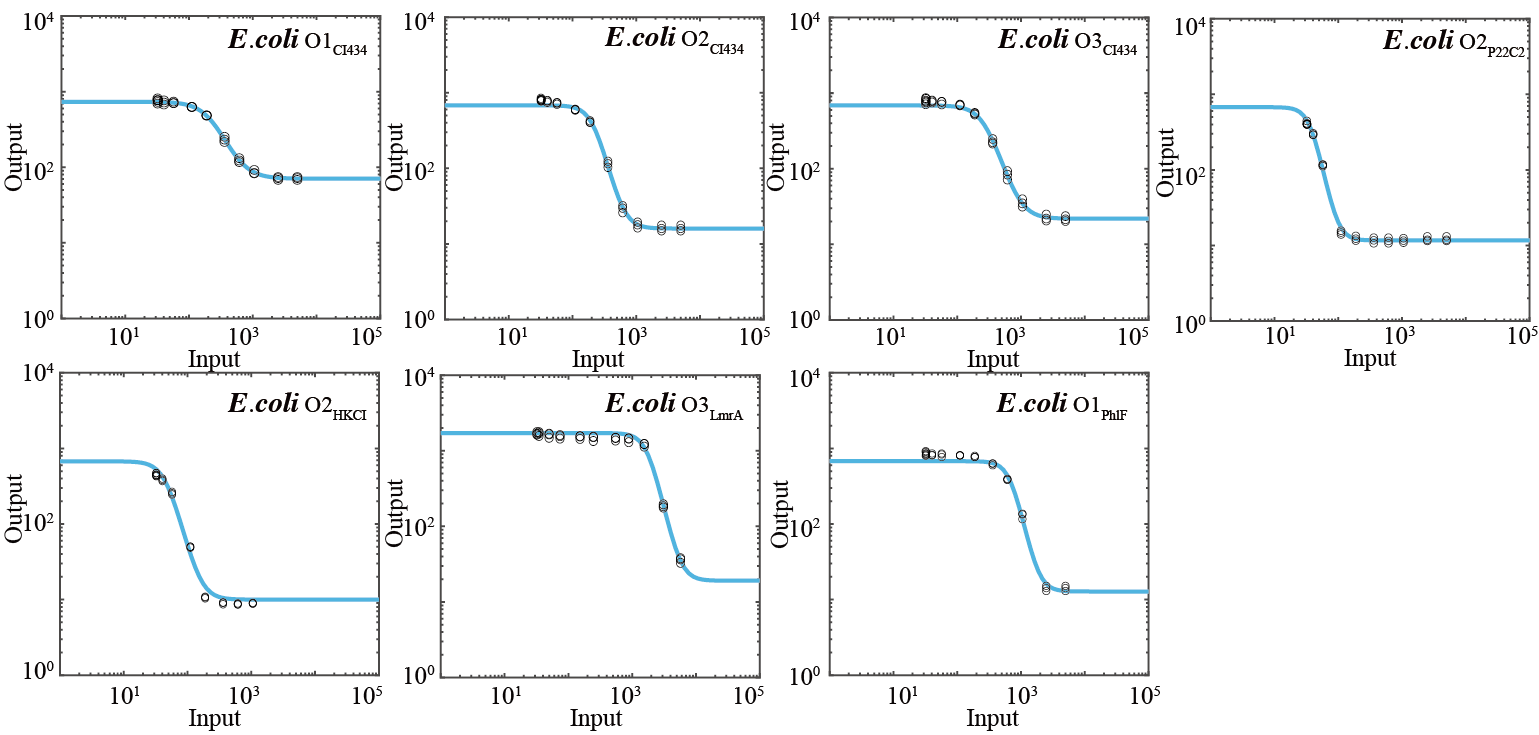


**Supplementary Figure 3. Experimental measurements and parameterization of transcriptional repressors.** Each repressor-promoter pair was converted to a NOT gate and characterized in *E. coli.* Error bars show the standard deviation of three independent replicates.


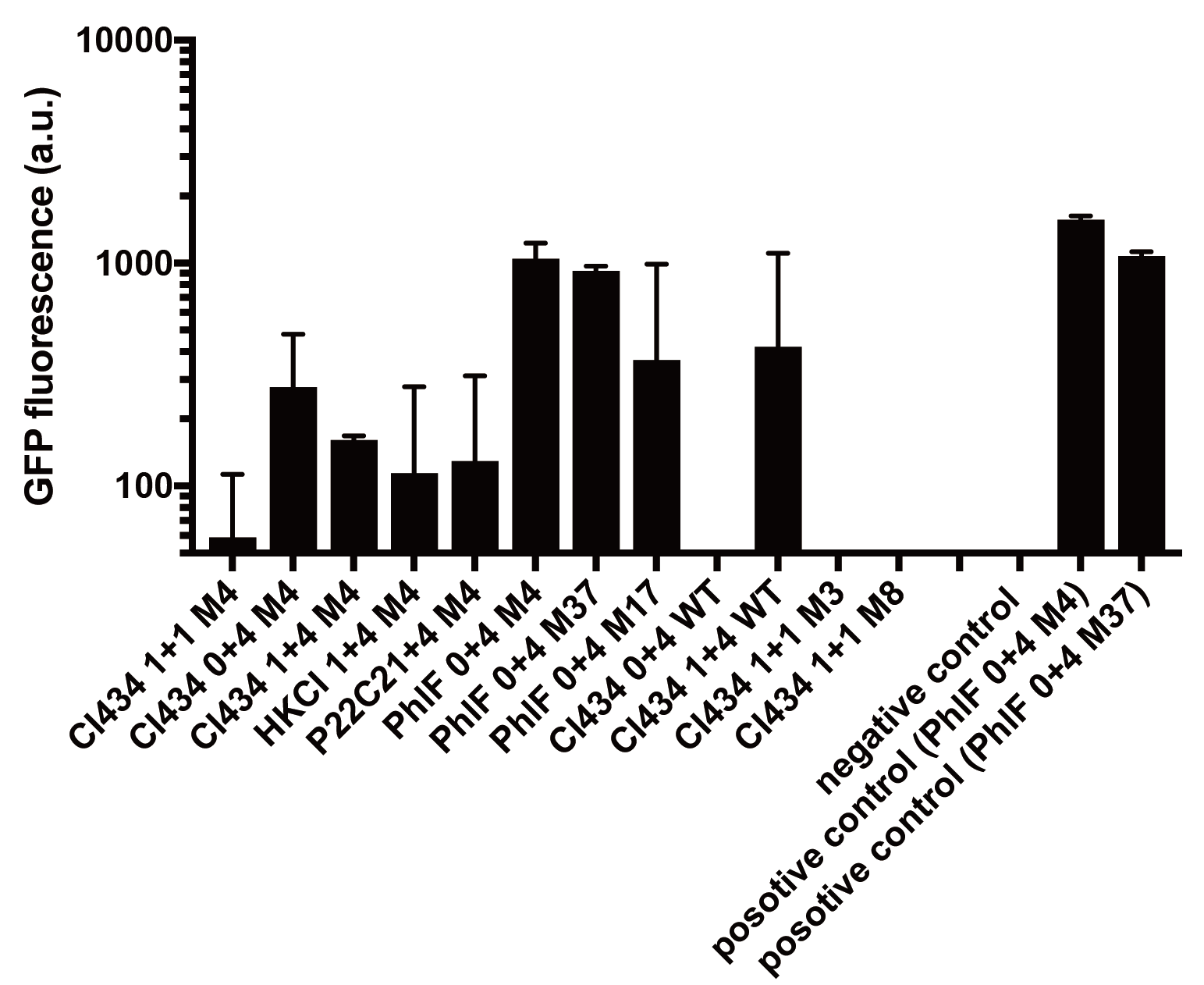


**Supplementary Figure 4. Spontaneous transcriprional activity of the synthetic promoters.** Error bars show the standard deviation of three independent replicates.

**
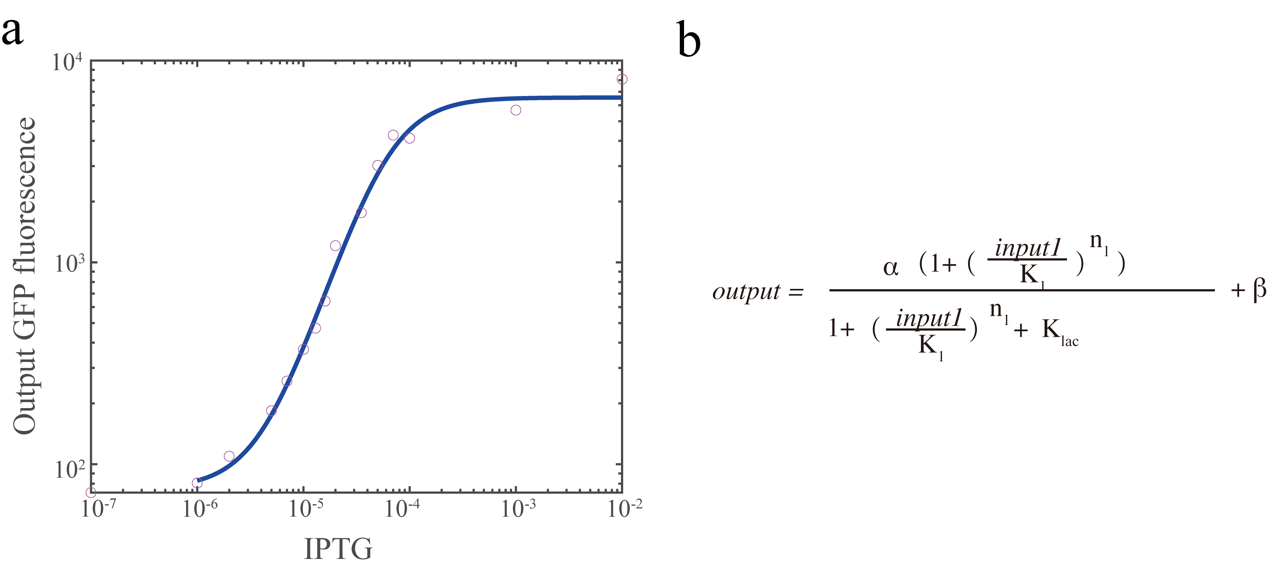
**

**Supplementary Figure 5. Characterization of an IPTG-inducible promoter. (a)** Response function of the P_TAC_ promoter using IPTG concentrations as input. Open circles represent experimental data. The solid blue curve represents the fitted results according to the biophysical model in **(b)**. The biophysical model was used to fit the response functions of P_TAC_ promoter variants. *K_1_* and *n_1_* represent the dissociation constants for IPTG and LacI, respectively; *α* and *β* denote the maximal and basal promoter activity, respectively; K_lac_ is the constant for LacI binding to lacO.


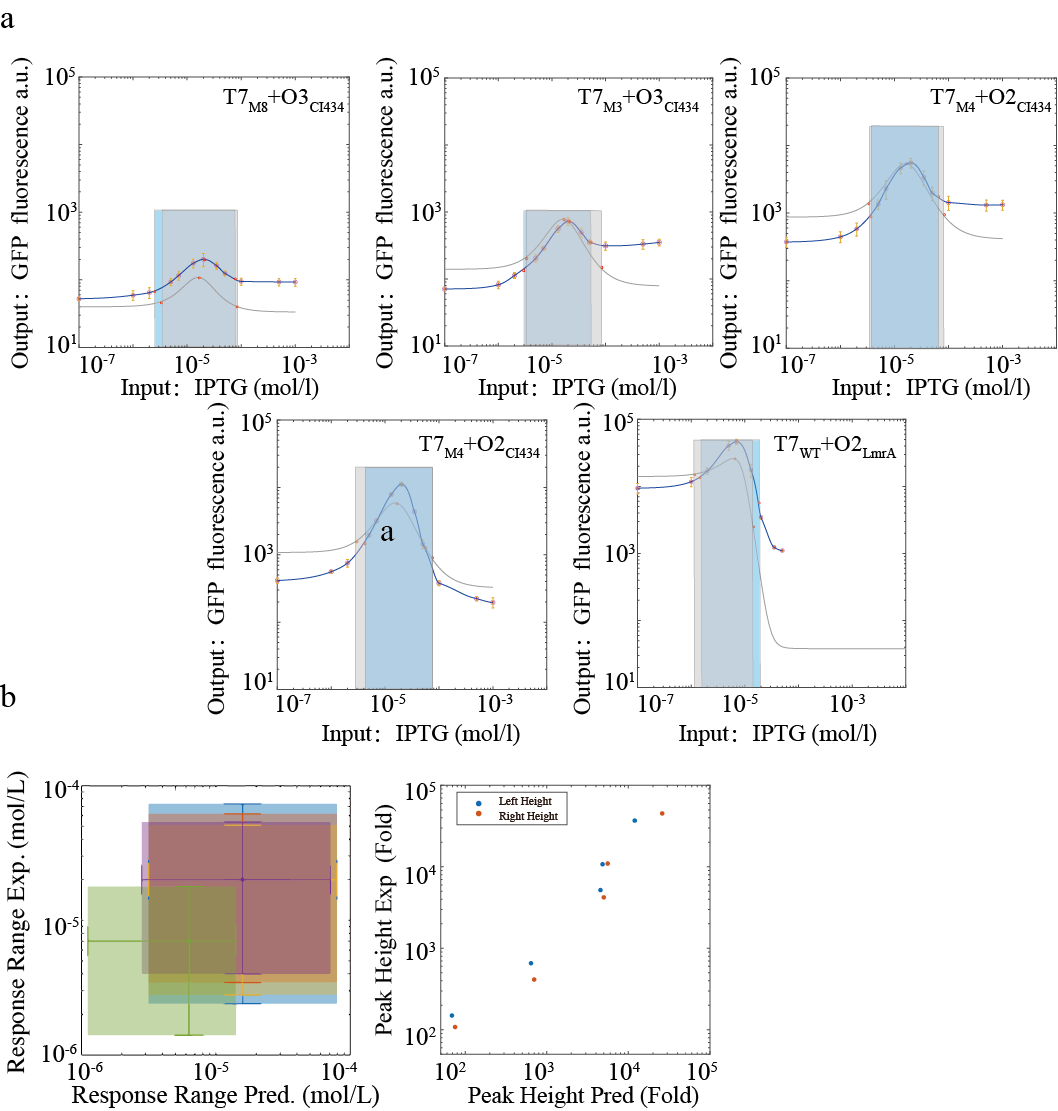


**Supplementary Figure 6. Experimentally measured and predicted response functions of IFF circuits in *C. glutamicum*. (a)** Experimental (orange circles and blue lines) and predicted (grey) response functions of the IFF circuits using PT7_M3_, PT7_M4_, PT7_M8_, and PT7wt as the core promoters. Error bars show the standard deviation of three independent replicates. The shaded intervals represent the response range, i.e., the input range where the output reached 10% of elevation from the left and from the right. (b) Comparison of the response range (left) and peak heights (right) between experimental measurement and model.

**
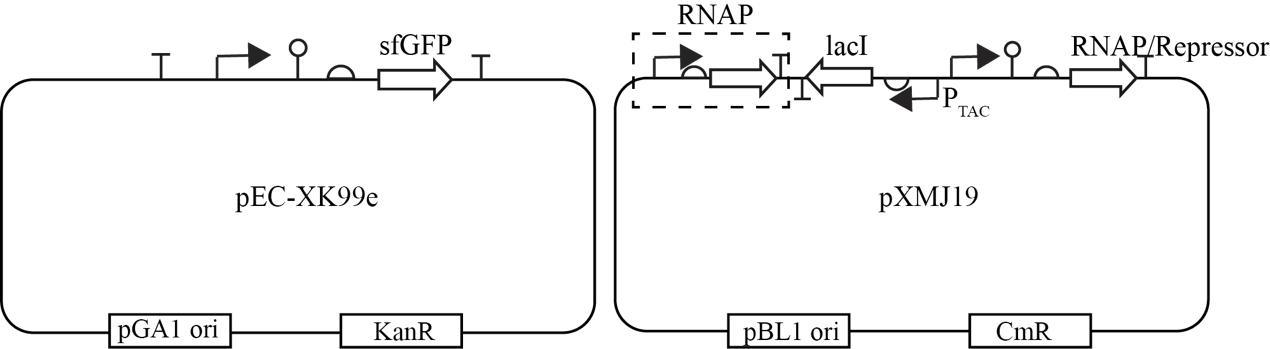
**

**Supplementary Figure 7. Plasmid Architecture of pXMJ19 and pEC-XK99e.** The vast majority of the plasmids used in *C. glutamicum* were derived from two basic vectors: pXMJ19 and pEC-XK99e. The corresponding sequences are summarized in Supplementary Tables 6-8, 12 and 13.

**Supplementary table 1. The T7 promoter library sequences used in this study.**

| M11 | \| CTAAA \| CGACTCA \| CTATA \| \| --- \| --- \| --- \| |
| --- | --- | --- | --- | --- |
| M12 | \| AAAAA \| CGACTCA \| CTATA \| \| --- \| --- \| --- \| |
| M13 | \| AGATA \| CGACTCA \| CTATA \| \| --- \| --- \| --- \| |
| M14 | \| GTAAA \| CGACTCA \| CTATA \| \| --- \| --- \| --- \| |
| M15 | \| TGATA \| CGACTCA \| CTATA \| \| --- \| --- \| --- \| |
| M16 | \| ATTTA \| CGACTCA \| CTATA \| \| --- \| --- \| --- \| |
| M17 | \| GAATA \| CGACTCA \| CTATA \| \| --- \| --- \| --- \| |
| M18 | \| GATTA \| CGACTCA \| CTATA \| \| --- \| --- \| --- \| |
| M19 | \| CAAAA \| CGACTCA \| CTATA \| \| --- \| --- \| --- \| |
| M20 | \| ACTTA \| CGACTCA \| CTATA \| \| --- \| --- \| --- \| |
| M21 | \| TCCTA \| CGACTCA \| CTATA \| \| --- \| --- \| --- \| |
| M22 | \| GCCTA \| CGACTCA \| CTATA \| \| --- \| --- \| --- \| |
| M23 | \| GTGAA \| CGACTCA \| CTATA \| \| --- \| --- \| --- \| |
| M24 | \| ACCTA \| CGACTCA \| CTATA \| \| --- \| --- \| --- \| |
| M25 | \| TTGAA \| CGACTCA \| CTATA \| \| --- \| --- \| --- \| |
| M26 | \| CACAA \| CGACTCA \| CTATA \| \| --- \| --- \| --- \| |
| M27 | \| ATAGA \| CGACTCA \| CTATA \| \| --- \| --- \| --- \| |
| M28 | \| CGCAA \| CGACTCA \| CTATA \| \| --- \| --- \| --- \| |
| M29 | \| CGCAC \| CGACTCA \| CTATA \| \| --- \| --- \| --- \| |
| M30 | \| TGCAG \| CGACTCA \| CTATA \| \| --- \| --- \| --- \| |
| M31 | \| GGTCG \| CGACTCA \| CTATA \| \| --- \| --- \| --- \| |
| M32 | \| CTACA \| CGACTCA \| CTATA \| \| --- \| --- \| --- \| |
| M33 | \| ATAAG \| CGACTCA \| CTATA \| \| --- \| --- \| --- \| |
| M34 | \| TTTGT \| CGACTCA \| CTATA \| \| --- \| --- \| --- \| |
| M35 | \| TATCG \| CGACTCA \| CTATA \| \| --- \| --- \| --- \| |
| M36 | \| CACTA \| CGACTCA \| CTATA \| \| --- \| --- \| --- \| |
| M37 | \| AAAGC \| CGACTCA \| CTATA \| \| --- \| --- \| --- \| |
| M38 | \| GCATT \| CGACTCA \| CTATA \| \| --- \| --- \| --- \| |
| M39 | \| CTCTG \| CGACTCA \| CTATA \| \| --- \| --- \| --- \| |
| M40 | \| AGCCA \| CGACTCA \| CTATA \| \| --- \| --- \| --- \| |
| M41 | \| AATTC \| CGACTCA \| CTATA \| \| --- \| --- \| --- \| |
| M42 | \| TTTAA \| CGACTCA \| CTATA \| \| --- \| --- \| --- \| |
| M43 | \| CTCAT \| CGACTCA \| CTATA \| \| --- \| --- \| --- \| |
| M44 | \| CCGTT \| CGACTCA \| CTATA \| \| --- \| --- \| --- \| |
| M45 | \| CGAAA \| CGACTCA \| CTATA \| \| --- \| --- \| --- \| |
| M46 | \| AGGAA \| CGACTCA \| CTATA \| \| --- \| --- \| --- \| |
| M47 | \| CATCA \| CGACTCA \| CTATA \| \| --- \| --- \| --- \| |
| M48 | \| GACAA \| CGACTCA \| CTATA \| \| --- \| --- \| --- \| |
| M49 | \| AGAAA \| CGACTCA \| CTATA \| \| --- \| --- \| --- \| |
| M51 | \| GGCTA \| CGACTCA \| CTATA \| \| --- \| --- \| --- \| |
| M50 | \| AATCA \| CGACTCA \| CTATA \| \| --- \| --- \| --- \| |
| M52 | \| AGTTA \| CGACTCA \| CTATA \| \| --- \| --- \| --- \| |
| M53 | \| AAATA \| CGACTCA \| CTATA \| \| --- \| --- \| --- \| |
| M54 | \| TAAAA \| CGACTCA \| CTATA \| \| --- \| --- \| --- \| |
| M55 | \| ATAAA \| CGACTCA \| CTATA \| \| --- \| --- \| --- \| |
| M56 | \| ATCTA \| CGACTCA \| CTATA \| \| --- \| --- \| --- \| |
| M57 | \| ACAAA \| CGACTCA \| CTATA \| \| --- \| --- \| --- \| |
| M58 | \| ATACA \| CGACTCA \| CTATA \| \| --- \| --- \| --- \| |
| M59 | \| TAAAC \| CGACTCA \| CTATA \| \| --- \| --- \| --- \| |
| WT | \| TAATA \| CGACTCA \| CTATA \| \| --- \| --- \| --- \| |
| M2 | \| GTGCA \| CGACTCA \| CTATA \| \| --- \| --- \| --- \| |
| M3 | \| GGATA \| CGACTCA \| CTATA \| \| --- \| --- \| --- \| |
| M4 | \| TATAA \| CGACTCA \| CTATA \| \| --- \| --- \| --- \| |
| M5 | \| TAATA \| CGACTCA \| CACTC \| \| --- \| --- \| --- \| |
| M8 | \| TAATA \| CGACTCA \| GTCAA \| \| --- \| --- \| --- \| |
| M9 | \| GAAAA \| CGACTCA \| CTATA \| \| --- \| --- \| --- \| |

**Supplementary Table 2. Fitted parameter values of the activation curves across species.**

| Fitted parameters  *E. coli* |  |  |  |
| --- | --- | --- | --- |
| n_A_ | 1.259 | 1.23 | 1.29 |
| K_A_ | 3000 | 2633.25 | 3462.17 |
| α | 8742 | 7952.15 | 9703.57 |
| β | 55.58 | 53.62 | 57.56 |
| Fitted parameters *C. glutamicum* |  |  |  |
| n_A_ | 1.28 | 1.22 | 1.45 |
| K_A_ | 1.60E+04 | 6.62E+03 | 1.84E+04 |
| α | 1.00E+05 | 4.66E+04 | 1.00E+05 |
| β | 20.21 | 1 | 74.2331 |
| Fitted parameters  *P. entomophila* |  |  |  |
| n_A_ | 1.385 | 1.3032971 | 1.4668544 |
| K_A_ | 1.79E+04 | 12382.669 | 32706.49 |
| α | 1.88E+04 | 13539.404 | 33845.361 |
| β | 17.37 | 15.561222 | 19.04166 |

**Supplementary Table 3. Fitted parameter values of the activator with different T7 promoter cores.**

|  | α | K_A_ | n_A_ | β |
| --- | --- | --- | --- | --- |
| WT | 11000 | 1338.24 | 1.36 | 7.21 |
| M3 |  | 15682.17 | 1.12 | 15.92 |
| M4 |  | 4072.78 | 1.21 | 53.27 |
| M8 |  | 23029.36 | 1.11 | 12.29 |
| M9 |  | 4994.08 | 1.19 | 12.74 |
|  | α | K_A_ | n_A_ | β |
| WT | 76700 | 678.05 | 1.37 | 29.28 |
| M3 |  | 13658.03 | 1.27 |  |
| M4 |  | 1993.33 | 1.50 |  |
| M8 |  | 54776.90 | 1.30 |  |
| M9 |  | 2867.61 | 1.48 |  |

**Supplementary Table 4. Fitted parameters of compositional promoters in *C. glutamicum* and *E. coli.***

| *C. glu* O1_CI434_ |  |  |  | *E. coli* O1_CI434_ |  |  |  |
| --- | --- | --- | --- | --- | --- | --- | --- |
| n_R_ | 2.41 | 2.24 | 2.65 | n_R_ | 2.47 | 2.31 | 2.63 |
| K_R_ | 221.3 | 211.67 | 236.9 | K_R_ | 222.5 | 212.9 | 232.2 |
| Β | 68.55 | 64.65 | 74.39 | Β | 70.64 | 67.84 | 73.45 |
| *C. glu* O2_CI434_ |  |  |  | *E. coli* O2_CI434_ |  |  |  |
| n_R_ | 2.90 | 2.65 | 3.26 | n_R_ | 3.67 | 3.29 | 4.04 |
| K_R_ | 535.4 | 511.42 | 564.12 | K_R_ | 214.1 | 199.8 | 228.3 |
| Β | 21.39 | 19.88 | 23.30 | Β | 15.97 | 14.65 | 17.29 |
| *C. glu* O3_CI434_ |  |  |  | *E. coli* O3_CI434_ |  |  |  |
| n_R_ | 2.561 | 2.31 | 2.89 | n_R_ | 2.98 | 2.66 | 3.29 |
| K_R_ | 532.2 | 502.89 | 566 | K_R_ | 274.9 | 254.8 | 295 |
| Β | 27.91 | 26.06 | 29.93 | Β | 21.81 | 19.76 | 23.87 |
| *C. glu* O2_P22C2_ |  |  |  | *E. coli* O2_P22C2_ |  |  |  |
| n_R_ | 4.878 | 4.51 | 5.36 | n_R_ | 4.41 | 4.09 | 4.73 |
| K_R_ | 163.7 | 158.53 | 171.25 | K_R_ | 36.97 | 35.82 | 38.13 |
| Β | 48.48 | 46.77 | 51.77 | Β | 11.67 | 11.13 | 12.21 |
| *C. glu* O2_HKCI_ |  |  |  | *E. coli* O2_HKCI_ |  |  |  |
| n_R_ | 3.89 | 3.65 | 4.22 | n_R_ | 3.5 | 3.11 | 3.89 |
| K_R_ | 185.1 | 180.67 | 193.04 | K_R_ | 45.61 | 41.92 | 49.31 |
| Β | 36.69 | 35.52 | 38.78 | Β | 10 |  |  |
| *C. glu* O3_LmrA_ |  |  |  | *E. coli* O3_LmrA_ |  |  |  |
| n_R_ | 6.27 | 5.73 | 6.75 | n_R_ | 4.0 | 3.37 | 4.63 |
| K_R_ | 203.4 | 192.95 | 209.12 | K_R_ | 1716 | 1587 | 1846 |
| Β | 38.47 | 32.69 | 41.64 | Β | 19.1 | 8.89 | 29.31 |
| *C. glu* O1_PhlF_ |  |  |  | *E. coli* O1_PhlF_ |  |  |  |
| n_R_ | 5.492 | 5.06 | 6.20 | n_R_ | 4.16 | 3.25 | 5.07 |
| K_R_ | 174.3 | 169.69 | 184.16 | K_R_ | 692.8 | 627.2 | 758.3 |
| β | 44.05 | 43.01 | 49.33 | β | 12.8 | 10.26 | 15.33 |

**Supplementary Table 5. Fitted parameters for the inducible promoters.**

| Parameters of P_TAC_ | | | | |
| --- | --- | --- | --- | --- |
| alpha | beta | K_1_ | n_1_ | K_1ac1_ |
| 6480.20 | 75.68 | 0.0000001 | 1.65 | 58706.86 |

**Supplementary Table 6. Part Sequences of the T7 activator and the repressors.**

| T7RNAP | ATGAACACGATTAACATCGCTAAGAACGACTTCTCTGACATCGAACTGGCTGCTATCCCGTTCAACACTCTGGCTGACCATTACGGTGAGCGTTTAGCTCGCGAACAGTTGGCCCTTGAGCATGAGTCTTACGAGATGGGTGAAGCACGCTTCCGCAAGATGTTTGAGCGTCAACTTAAAGCTGGTGAGGTTGCGGATAACGCTGCCGCCAAGCCTCTCATCACTACCCTACTCCCTAAGATGATTGCACGCATCAACGACTGGTTTGAGGAAGTGAAAGCTAAGCGCGGCAAGCGCCCGACAGCCTTCCAGTTCCTGCAAGAAATCAAGCCGGAAGCCGTAGCGTACATCACCATTAAGACCACTCTGGCTTGCCTAACCAGTGCTGACAATACAACCGTTCAGGCTGTAGCAAGCGCAATCGGTCGGGCCATTGAGGACGAGGCTCGCTTCGGTCGTATCCGTGACCTTGAAGCTAAGCACTTCAAGAAAAACGTTGAGGAACAACTCAACAAGCGCGTAGGGCACGTCTACAAGAAAGCATTTATGCAAGTTGTCGAGGCTGACATGCTCTCTAAGGGTCTACTCGGTGGCGAGGCGTGGTCTTCGTGGCATAAGGAAGACTCTATTCATGTAGGAGTACGCTGCATCGAGATGCTCATTGAGTCAACCGGAATGGTTAGCTTACACCGCCAAAATGCTGGCGTAGTAGGTCAAGACTCTGAGACTATCGAACTCGCACCTGAATACGCTGAGGCTATCGCAACCCGTGCAGGTGCGCTGGCTGGCATCTCTCCGATGTTCCAACCTTGCGTAGTTCCTCCTAAGCCGTGGACTGGCATTACTGGTGGTGGCTATTGGGCTAACGGTCGTCGTCCTCTGGCGCTGGTGCGTACTCACAGTAAGAAAGCACTGATGCGCTACGAAGACGTTTACATGCCTGAGGTGTACAAAGCGATTAACATTGCGCAAAACACCGCATGGAAAATCAACAAGAAAGTCCTAGCGGTCGCCAACGTAATCACCAAGTGGAAGCATTGTCCGGTCGAGGACATCCCTGCGATTGAGCGTGAAGAACTCCCGATGAAACCGGAAGACATCGACATGAATCCTGAGGCTCTCACCGCGTGGAAACGTGCTGCCGCTGCTGTGTACCGCAAGGACAAGGCTCGCAAGTCTCGCCGTATCAGCCTTGAGTTCATGCTTGAGCAAGCCAATAAGTTTGCTAACCATAAGGCCATCTGGTTCCCTTACAACATGGACTGGCGCGGTCGTGTTTACGCTGTGTCAATGTTCAACCCGCAAGGTAACGATATGACCAAAGGACTGCTTACGCTGGCGAAAGGTAAACCAATCGGTAAGGAAGGTTACTACTGGCTGAAAATCCACGGTGCAAACTGTGCGGGTGTCGATAAGGTTCCGTTCCCTGAGCGCATCAAGTTCATTGAGGAAAACCACGAGAACATCATGGCTTGCGCTAAGTCTCCACTGGAGAACACTTGGTGGGCTGAGCAAGATTCTCCGTTCTGCTTCCTTGCGTTCTGCTTTGAGTACGCTGGGGTACAGCACCACGGCCTGAGCTATAACTGCTCCCTTCCGCTGGCGTTTGACGGGTCTTGCTCTGGCATCCAGCACTTCTCCGCGATGCTCCGAGATGAGGTAGGTGGTCGCGCGGTTAACTTGCTTCCTAGTGAAACCGTTCAGGACATCTACGGGATTGTTGCTAAGAAAGTCAACGAGATTCTACAAGCAGACGCAATCAATGGGACCGATAACGAAGTAGTTACCGTGACCGATGAGAACACTGGTGAAATCTCTGAGAAAGTCAAGCTGGGCACTAAGGCACTGGCTGGTCAATGGCTGGCTTACGGTGTTACTCGCAGTGTGACTAAGCGTTCAGTCATGACGCTGGCTTACGGGTCCAAAGAGTTCGGCTTCCGTCAACAAGTGCTGGAAGATACCATTCAGCCAGCTATTGATTCCGGCAAGGGTCTGATGTTCACTCAGCCGAATCAGGCTGCTGGATACATGGCTAAGCTGATTTGGGAATCTGTGAGCGTGACGGTGGTAGCTGCGGTTGAAGCAATGAACTGGCTTAAGTCTGCTGCTAAGCTGCTGGCTGCTGAGGTCAAAGATAAGAAGACTGGAGAGATTCTTCGCAAGCGTTGCGCTGTGCATTGGGTAACTCCTGATGGTTTCCCTGTGTGGCAGGAATACAAGAAGCCTATTCAGACGCGCTTGAACCTGATGTTCCTCGGTCAGTTCCGCTTACAGCCTACCATTAACACCAACAAAGATAGCGAGATTGATGCACACAAACAGGAGTCTGGTATCGCTCCTAACTTTGTACACAGCCAAGACGGTAGCCACCTTCGTAAGACTGTAGTGTGGGCACACGAGAAGTACGGAATCGAATCTTTTGCACTGATTCACGACTCCTTCGGTACCATTCCGGCTGACGCTGCGAACCTGTTCAAAGCAGTGCGCGAAACTATGGTTGACACATATGAGTCTTGTGATGTACTGGCTGATTTCTACGACCAGTTCGCTGACCAGTTGCACGAGTCTCAATTGGACAAAATGCCAGCACTTCCGGCTAAAGGTAACTTGAACCTCCGTGACATCTTAGAGTCGGACTTCGCGTTCGCGTAA |
| --- | --- |
| CI434 | ATGAGTATTTCTTCCAGGGTAAAAAGCAAAAGAATCCAGCTTGGACTTAACCAGGCTGAACTTGCTCAAAAGGTGGGGACTACCCAGCAGTCTATAGAGCAGCTCGAAAACGGTAAAACTAAGCGACCACGCTTTTTACCAGAACTTGCGTCAGCTCTTGGCGTAAGTGTTGACTGGCTGCTCAATGGCACCTCTGATTCGAATGTTAGATTTGTTGGGCACGTTGAGCCCAAAGGGAAATATCCATTGATTAGCATGGTTAGAGCTGGTTCGTGGTGTGAAGCTTGTGAACCCTACGATATCAAGGACATTGATGAATGGTATGACAGTGACGTTAACTTATTAGGCAATGGATTCTGGCTGAAGGTTGAAGGTGATTCCATGACCTCACCTGTAGGTCAAAGCATCCCTGAAGGTCATATGGTGTTAGTAGATACTGGACGGGAGCCAGTGAATGGAAGCCTTGTTGTAGCCAAACTGACTGACGCGAACGAAGCAACATTCAAGAAACTGGTCATAGATGGCGGTCAGAAGTACCTGAAAGGCCTGAATCCTTCATGGCCTATGACTCCTATCAACGGAAACTGCAAGATTATCGGTGTTGTCGTGGAAGCGAGGGTAAAATTCGTATA |
| HKCI | ATGGTTCAACAGAAAGAGCGTGAAACTTTCTCGCAGAGGCTTGCGCTGGCCTGTGATAAAGCGGGATTACCTTTGCATGGTAGGCAGGCTGATTTAGCTGTCAGGCTTAAGGTCACACCAAAAGCCATTAGTAAATGGTTCAACGGGGAGTCAATACCAAGAAAAGACAAGATGGAATCTCTGGCTTCGGTGCTGGGAACTACTGCTGCATATCTGCATGGCTATGCTGATGATGACGGTATCACGGTAAATCATCTATCAAGATCAAATGATTATTATCGTGTTGATGTATTGGATGTTCAGGCGAGCGCCGGGCCAGGAACCATGGTTTCCAATGAATTTATAGAAAAGATAAGAGCAATTGAATATACGACCGAGCAGGCAAGAATTTTATTTAATGGAAGGCCACAGGAAAGCGTAAAAGTCATCACGGTTCGCGGTGACAGCATGGAGGGAACCATCAATCCGGGAGATGAGATCTTTGTTGATGTATCCATAACCTGTTTTGATGGCGATGGCATTTATGTGTTTGTATACGGGAAAACAATGCACGTTAAGCGCCTGCAAATGCAAAAGAACAGGCTTGCCGTCATCTCTGACAATGCCGCTTATGATCGATGGTACATAGAAGAAGGTGAAGAAGAGCAACTTCACATTCTAGCCAAAGTCCTCATTAGGCAGTCAATCGATTACAAGCGATTCGGA |
| P22C2 | ATGGGTGAGCGTATTCGCGCTCGAAGAAAAAAACTCAAGATTAGACAAGCCGCTCTTGGTAAGATGGTGGGAGTGTCTAATGTTGCAATATCGCAATGGGAGCGCTCGGAGACTGAGCCAAATGGGGAGAACCTGTTGGCACTTTCGAAGGCTCTTCAGTGCTCCCCTGACTATTTGCTGAAAGGAGATTTAAGCCAGACAAACGTTGCCTATCATAGTAGGCATGAGCCAAGAGGATCATACCCTCTTATCAGTTGGGTAAGCGCAGGGCAATGGATGGAAGCTGTAGAACCTTATCACAAGCGCGCGATAGAGAACTGGCACGACACCACTGTAGATTGTTCAGAAGATTCATTTTGGCTTGATGTCCAAGGTGACTCTATGACAGCACCGGCAGGGTTAAGCATTCCAGAAGGAATGATAATTCTGGTTGATCCCGAAGTCGAACCAAGAAACGGCAAGCTGGTTGTTGCAAAATTAGAAGGTGAAAACGAGGCCACATTCAAAAAATTAGTTATGGATGCAGGCCGAAAGTTTTTAAAACCATTAAACCCACAATATCCGATGATAGAAATCAACGGAAACTGCAAAATCATTGGCGTAGTTGTTGACGCAAAACTCGCAAATCTTCCATAG |
| PhlF | ATGGCACGTACCCCGAGCCGTAGCAGCATTGGTAGCCTGCGTAGTCCGCATACCCATAAAGCAATTCTGACCAGCACCATTGAAATCCTGAAAGAATGTGGTTATAGCGGTCTGAGCATTGAAAGCGTTGCACGTCGTGCCGGTGCAAGCAAACCGACCATTTATCGTTGGTGGACCAATAAAGCAGCACTGATTGCCGAAGTGTATGAAAATGAAAGCGAACAGGTGCGTAAATTTCCGGATCTGGGTAGCTTTAAAGCCGATCTGGATTTTCTGCTGCGTAATCTGTGGAAAGTTTGGCGTGAAACCATTTGTGGTGAAGCATTTCGTTGTGTTATTGCAGAAGCACAGCTGGACCCTGCAACCCTGACCCAGCTGAAAGATCAGTTTATGGAACGTCGTCGTGAGATGCCGAAAAAACTGGTTGAAAATGCCATTAGCAATGGTGAACTGCCGAAAGATACCAATCGTGAACTGCTGCTGGATATGATTTTTGGTTTTTGTTGGTATCGCCTGCTGACCGAACAGCTGACCGTTGAACAGGATATTGAAGAATTTACCTTCCTGCTGATTAATGGTGTTTGTCCGGGTACACAGCGTTGA |
| LmrA | ATGAGCTATGGTGATAGCCGTGAAAAAATTCTGAGCGCAGCAACCCGTCTGTTTCAGCTGCAGGGTTATTATGGCACCGGTCTGAATCAGATTATCAAAGAAAGCGGTGCACCGAAAGGTAGCCTGTATTATCATTTTCCGGGTGGTAAAGAACAGCTGGCAATTGAAGCAGTGAACGAAATGAAAGAATATATCCGCCAGAAAATCGCCGATTGTATGGAAGCATGTACCGATCCGGCAGAAGGTATTCAGGCATTTCTGAAAGAACTGAGCTGTCAGTTTAGCTGTACCGAAGATATTGAAGGTCTGCCGGTTGGTCTGCTGGCAGCAGAAACCAGCCTGAAAAGCGAACCGCTGCGTGAAGCATGTCATGAAGCATATAAAGAATGGGCCAGCGTGTATGAAGAAAAACTGCGTCAGACCGGTTGTAGCGAAAGCCGTGCAAAAGAAGCAAGCACCGTTGTTAATGCAATGATTGAAGGTGGTATTCTGCTGAGCCTGACCGCAAAAAATAGCACACCGCTGCTGCATATTAGCAGCTGTATTCCGGATCTGCTGAAACGT |

**Supplementary Table 7. Sequences of promoter cores and operators. Operator sequences are highlighted in yellow.**

| pT7M4-O1_CI434_ | TATAACGACTCACTATAGGGGTACAAGAAAGTTTGTTgctacTACAAGAAAGTTTGTTgctacTACAAGAAAGTTTGTTgctacTACAAGAAAGTTTGTT |
| --- | --- |
| pT7M4-O2_CI434_ | TACAAGAAAGTTTGTTctatgTATAACGACTCACTATAGGGGTACAAGAAAGTTTGTTgctacTACAAGAAAGTTTGTTgctacTACAAGAAAGTTTGTTgctacTACAAGAAAGTTTGTT |
| pT7M4-O3_CI434_ | TACAAGAAAGTTTGTTctatgTATAACGACTCACTATAGGGGTACAAGAAAGTTTGTT |
| pT7M4-O2_HKCI_ | TGAACCATAAGTTCAgctatgTATAACGACTCACTATAggggTGAACCATAAGTTCAgctctgTGAACCATAAGTTCAgctctgTGAACCATAAGTTCAgctctgTGAACCATAAGTTCA |
| pT7M4-O2_P22C2_ | ATTTAAGTGTTCTTTAATgagcatctgctatgTATAACGACTCACTATAggggATTTAAGTGTTCTTTAATcgctgttccgctgATTTAAGTGTTCTTTAATcgctgttccgctgATTTAAGTGTTCTTTAATcgctgttccgctgATTTAAGTGTTCTTTAAT |
| pT7WT-O3_LmrA_ | GATAATAGACCAGTCACTATATTTtagctactcaacctatgTAATACGACTCACTATAggggGATAATAGACCAGTCACTATATTTtagctac |
| pT7M4-O1_PhlF_ | TATAACGACTCACTATAggggATGATACGAAACGTACCGTATCGTTAAGGTcATGATACGAAACGTACCGTATCGTTAAGGTcATGATACGAAACGTACCGTATCGTTAAGGTcATGATACGAAACGTACCGTATCGTTAAGGTc |

**Supplementary Table 8. The plasmids used in *C. glutamicum*** **in this study.**

| Experiment | | Plasmid 1  (**Cmr-pBL1 ori)** | Plasmid 2  (**KanR-pGA1 ori)** | Inducer |
| --- | --- | --- | --- | --- |
| Promoter library | | P_A6_-RNAP | pEC-XK99e-promoter library | none |
| Transcriptional activation | Input curve | P_TAC_-sfGFP | pEC-XK99e | IPTG gradient |
|  | Output curve | P_TAC_-RNAP | pEC-XK99e-promoter core | IPTG gradient |
| Transcriptional repression | Input curve | P_TAC_- sfGFP | pEC-XK99e | IPTG gradient |
|  | Output curve | P_A6_-RNAP- P_TAC_-Repressor | pEC-XK99e-promoter-operator | IPTG gradient |

**Supplementary Table 9. The plasmids used in *E. coli*** **in this study.**

| Experiment | | Plasmid 1  (**Ampr-p15A ori)** | Plasmid 2  **(Cmr-pSC101 ori)** | Inducer |
| --- | --- | --- | --- | --- |
| Promoter library | | P_SAL_ -RNAP | pPT -promoter library | none |
| Transcriptional activation | Input curve | P_TAC_-sfGFP | pPT | IPTG gradient |
|  | Output curve | P_TAC_-RNAP | pPT -promoter core | IPTG gradient |
| Transcriptional repression | Input curve | P_TAC_- sfGFP | pPT | IPTG gradient |
|  | Output curve | P_SAL_ -RNAP- P_TAC_-Repressor | pPT -promoter-operator | IPTG gradient |

**Supplementary Table 10. The plasmids used in *P. entemophila*** **in this study.**

| Experiment | | Plasmid 1  (**Ampr-p15A ori)** | Plasmid 2  **(Cmr-pSC101 ori)** | Inducer |
| --- | --- | --- | --- | --- |
| Promoter library | | P_SAL_ -RNAP | pPT -promoter library | none |
| Transcriptional activation | Input curve | P_TAC_-sfGFP | pPT | IPTG gradient |
|  | Output curve | P_TAC_-RNAP | pPT -promoter core | IPTG gradient |

**Supplementary Table 11. The plasmids used in *S. albus J1074*** **in this study.**

| Experiment | Plasmid 1  pSOK611  (**Ampr-ori ColE1)** | Plasmid 2  pIJ8660  (**Ampr-ori ColE1)** | Inducer |
| --- | --- | --- | --- |
| Promoter library | P21-RNAP(genomic integration) | pIJ8660  -promoter library | none |

**Supplementary Table 12. Sequences of promoters in different strains.**

| P_A6_-RBS  *C. glutamicum* | CAGAAAATTATTTTAAATTTCCTCTTGACACCCACAGGGACCTGAGGTACGGCCTAGTGCAGCTGTCACCGGATGTGCTTTCCGGTCTGATGAGTCCGTGAGGACGAAACAGCCTCTACAAATAATTTTGTTTAAtactagagtcacacaggaaagtactag |
| --- | --- |
| P_TAC_  *C. glutamicum*  Constitutively expressed lacI c (in reverse orientation) | TCACTGCCCGCTTTCCAGTCGGGAAACCTGTCGTGCCA GCTGCATTAATGAATCGGCCAACGCGCGGGGAGAGGC GGTTTGCGTATTGGGCGCCAGGGTGGTTTTTCTTTTCA CCAGTGAGACTGGCAACAGCTGATTGCCCTTCACCGC CTGGCCCTGAGAGAGTTGCAGCAAGCGGTCCACGCTG GTTTGCCCCAGCAGGCGAAAATCCTGTTTGATGGTGGT TAACGGCGGGATATAACATGAGCTATCTTCGGTATCGTC GTATCCCACTACCGAGATATCCGCACCAACGCGCAGCC CGGACTCGGTAATGGCGCGCATTGCGCCCAGCGCCATC TGATCGTTGGCAACCAGCATCGCAGTGGGAACGATGC CCTCATTCAGCATTTGCATGGTTTGTTGAAAACCGGAC ATGGCACTCCAGTCGCCTTCCCGTTCCGCTATCGGCTG AATTTGATTGCGAGTGAGATATTTATGCCAGCCAGCCA GACGCAGACGCGCCGAGACAGAACTTAATGGGCCCGC TAACAGCGCGATTTGCTGGTGACCCAATGCGACCAGAT GCTCCACGCCCAGTCGCGTACCGTCCTCATGGGAGAA AATAATACTGTTGATGGGTGTCTGGTCAGAGACATCAA GAAATAACGCCGGAACATTAGTGCAGGCAGCTTCCAC AGCAATGGCATCCTGGTCATCCAGCGGATAGTTAATGA TCAGCCCACTGACGCGTTGCGCGAGAAGATTGTGCAC CGCCGCTTTACAGGCTTCGACGCCGCTTCGTTCTACCA TCGACACCACCACGCTGGCACCCAGTTGATCGGCGCG AGATTTAATCGCCGCGACAATTTGCGACGGCGCGTGCA GGGCCAGACTGGAGGTGGCAACGCCAATCAGCAACG ACTGTTTGCCCGCCAGTTGTTGTGCCACGCGGTTGGGA ATGTAATTCAGCTCCGCCATCGCCGCTTCCACTTTTTCC CGCGTTTTCGCAGAAACGTGGCTGGCCTGGTTCACCA CGCGGGAAACGGTCTGATAAGAGACACCGGCATACTC TGCGACATCGTATAACGTTACTGGTTTCATATTCACCAC CCTGAATTGACTCTCTTCCGGGCGCTATCATGCCATACC GCGAAAGGTTTTGCGCCATTCGATGGCGCGCCGCGCA CTCAACCATTGTAACTCAAAACTAAAAAAATGTCAATT CGTCAGGCCACATAGCTTTCTTGTTCTGATCGGAACGA TCGTTGGCTGTGTTGACAATTAATCATCGGCTCGTATAATGTGTGGAATTGTGAGCGCTCACAATTAGCTGTCACCGGATGTGCTTTCCGGTCTGATGAGTCCGTGAGGACGAAACAGCCTCTACAAATAATTTTGTTTAA |
| P_TAC_  *E. coli* | TCACTGCCCGCTTTCCAGTCGGGAAACCTGTCGTGCCA GCTGCATTAATGAATCGGCCAACGCGCGGGGAGAGGC GGTTTGCGTATTGGGCGCCAGGGTGGTTTTTCTTTTCA CCAGTGAGACTGGCAACAGCTGATTGCCCTTCACCGC CTGGCCCTGAGAGAGTTGCAGCAAGCGGTCCACGCTG GTTTGCCCCAGCAGGCGAAAATCCTGTTTGATGGTGGT TAACGGCGGGATATAACATGAGCTATCTTCGGTATCGTC GTATCCCACTACCGAGATATCCGCACCAACGCGCAGCC CGGACTCGGTAATGGCGCGCATTGCGCCCAGCGCCATC TGATCGTTGGCAACCAGCATCGCAGTGGGAACGATGC CCTCATTCAGCATTTGCATGGTTTGTTGAAAACCGGAC ATGGCACTCCAGTCGCCTTCCCGTTCCGCTATCGGCTG AATTTGATTGCGAGTGAGATATTTATGCCAGCCAGCCA GACGCAGACGCGCCGAGACAGAACTTAATGGGCCCGC TAACAGCGCGATTTGCTGGTGACCCAATGCGACCAGAT GCTCCACGCCCAGTCGCGTACCGTCCTCATGGGAGAA AATAATACTGTTGATGGGTGTCTGGTCAGAGACATCAA GAAATAACGCCGGAACATTAGTGCAGGCAGCTTCCAC AGCAATGGCATCCTGGTCATCCAGCGGATAGTTAATGA TCAGCCCACTGACGCGTTGCGCGAGAAGATTGTGCAC CGCCGCTTTACAGGCTTCGACGCCGCTTCGTTCTACCA TCGACACCACCACGCTGGCACCCAGTTGATCGGCGCG AGATTTAATCGCCGCGACAATTTGCGACGGCGCGTGCA GGGCCAGACTGGAGGTGGCAACGCCAATCAGCAACG ACTGTTTGCCCGCCAGTTGTTGTGCCACGCGGTTGGGA ATGTAATTCAGCTCCGCCATCGCCGCTTCCACTTTTTCC CGCGTTTTCGCAGAAACGTGGCTGGCCTGGTTCACCA CGCGGGAAACGGTCTGATAAGAGACACCGGCATACTC TGCGACATCGTATAACGTTACTGGTTTCATATTCACCAC CCTGAATTGACTCTCTTCCGGGCGCTATCATGCCATACC GCGAAAGGTTTTGCGCCATTCGATGGCGCGCCGCTTCG TCAGGCCACATAGCTTTCTTGTTCTGATCGGAACGATC GTTGGCTGtgttgacaattaatcatcggctcgtataatgtgtggaattgtgagcgctc acaattAGCTGTCACCGGATGTGCTTTCCGGTCTGATGAG TCCGTGAGGACGAAACAGCCTCTACAAATAATTTTGTT TAA |
| Psal  *E. coli* | GAATTCGCGGCCGCTTCTAGAGTCAATCCGTAAACAGGTCAAACATCAGTTGCCGCAACCAAATATTGGCTAGGTCCTTGTGGTACTTCGCATGCCAGAACATGTTGATGGCTATTTCAGGCAAGACGACTGGGTGCGGCAAGGCGCTTAGGCCGAAGGGCTCCACGCAGCAGTCGGCTAAACGTATCGGCACAGTGGCGAGCAGATCGGTGCGCTGGAGGATGTGGCCAACGGCGGCGAAGTGCGGCACTTCCAGACGGATGTCGCGCCGGATGCCGACCCGTGTCATGTACGTGTCCACCTCGCCGTGGCCGGTGCCAGCGGCGATGACACGCACGTGGCCGTAGGAACAGAAGCGCTCCAGAGTCAGGGGTTCGCGGGTGACTGGATGGTCCTTGCGACATAGGCACACGTAGTGATTCTGGAGCAGCCGGCGCTGAAAGAAGCCAGTTTGCAGATTGGGAAGCAGGCCCACGGCCAAGTCCACGGTTCCGTTCTGCAAGGCCTGCATCAGGCTCATCGAACTGTCGCGCACCGTACTGATCACGCAATTGGGGGCCTGGTGAGCCAGCACATCCATCAGCCGCGGCATGAAGTAGATCTCGCCAATGTCGGTCATGGCCAGGGTGAAGGTACGCTCGCTGGTCAGCGGATCGAAGCTTTCATGGTGCTGTAGGGCGTTGCGCAGTGCGTGCATGGCCGAAGTGACGGGCTCGGCCAGATGCGCGGCATAGGGTGTGGGTTCCATTCCCTGATGTGTGCGCACGAAGAGTGGGTCCTGTAGCGAGGTGCGCAGGCGTTTCAGCGCATTGCTCACGGCAGGCTGGGTCAGGCCCAGGTTCTCCGCAGTGATAGAGACGCGTCTGTCGACCAGCAACTGGTTGAACACCACCAGCAGGTTTAAATCCAGGTCACGCAGTTCCATGGGGCCTCGCTTGGGTTATTGCTGGTGCCCGGCCGGGCGCAATATTCATGTTGATGATTTATTATATATCGAGTGGTGTATTTATCAATATTGTTTGCTCCGTTATCGTTATTAACAAGTCATCAATAAAGCCATCACGAG |
| Pre-RBSM5  *P. entemophila* | TCGCCTATGCTCTGGGGCCTCGGCAGATGCGAGCGCTGCATACCGTCCGGTAGGTCGGGAAGCGTGCAGTGCCGAGGCGGATTCCCGCATTGACAGCGCGTGCGTTGCAAGGCAACAATGGACTCAAATGTCTCGGAATCGCTGACGATTCCCAGGTTTCTCCGGCAAGCATAGCGCATGGCGTCTCCATGCGAGAATGTCGCGCTTGCCGGATAAAAGGGGAGCCGCTATCGGAATGGACGCAAGCCACGGCCGCAGCAGGTGCGGTCGAGGGCTTCCAGCCAGTTCCAGGGCAGATGTGCCGGCAGACCCTCCCGCTTTGGGGGAGGCGCAAGCCGGGTCCATTCGGATAGCATCTCCCCATGCAAAGTGCCGGCCAGGGCAATGCCCGGAGCCAGCTGTCACCGGATGTGCTTTCCGGTCTGATGAGTCCGTGAGGACGAAACAGCCTCTACAAATAATTTTGTTtactaggggggttcttcctcgtactag |
| Plux-RBSM5  *P. entemophila*  Constitutively expressed lacI c (in reverse orientation) | ATGCCTCCACACCGCTCGTCACATCCTGAATTCTAAAGATCTGGTGAAACAAAACGGTAGACAACATGAAGTAAACAGGTACGATGTACCACATGAAACGACAGTGAGTCAATTAAAGAGGAGAAAGGTACCATGAAAAACATAAATGCCGACGACACATACAGAATAATTAATAAAATTAAAGCTTGTAGAAGCAATAATGATATTAATCAATGCTTATCTGATATGACTAAAATGGTACATTGTGAATATTATTTACTCGCGATCATTTATCCTCATTCTATGGTTAAATCTGATATTTCAATCCTAGATAATTACCCTAAAAAATGGAGGCAATATTATGATGACGCTAATTTAATAAAATATGATCCTATAGTAGATTATTCTAACTCCAATCATTCACCAATTAATTGGAATATATTTGAAAACAATGCTGTAAATAAAAAATCTCCAAATGTAATTAAAGAAGCGAAAACATCAGGTCTTATCACTGGGTTTAGTTTCCCTATTCATACGGCTAACAATGGCTTCGGAATGCTTAGTTTTGCACATTCAGAAAAAGACAACTATATAGATAGTTTATTTTTACATGCGTGTATGAACATACCATTAATTGTTCCTTCTCTAGTTGATAATTATCGAAAAATAAATATAGCAAATAATAAATCAAACAACGATTTAACCAAAAGAGAAAAAGAATGTTTAGCGTGGGCATGCGAAGGAAAAAGCTCTTGGGATATTTCAAAAATATTAGGTTGCAGTGAGCGTACTGTCACTTTCCATTTAACCAATGCGCAAATGAAACTCAATACAACAAACCGCTGCCAAAGTATTTCTAAAGCAATTTTAACAGGAGCAATTGATTGCCCATACTTTAAAAATTAATAATACTAGAGCTCGGTACCAAATTCCAGAAAAGAGACGCTTTCGAGCGTCTTTTTTCGTTTTGGTCCTACTAGCTCGGTACCTTCGTCAGGCCACATAGCTTTCTTGTTCTGATCGGAACGATCGTTGGCTGACCTGTAGGATCGTACAGGTTTACGCAAGAAAATGGTTTGTTACTTTCGAATAAAAGCTGTCACCGGATGTGCTTTCCGGTCTGATGAGTCCGTGAGGACGAAACAGCCTCTACAAATAATTTTGTTTAAtactaggggggttcttcctcgtactag |
| P21-CuO-RBS | TGTGCGGGCTCTAACACGTCCTAGTATGGTAGGATGAGCAAAACAAACAGACAATCTGGTCTGTTTGTATTATcacaaggggttgtgaccggg |
| T7RNAP sequence in *S. albus J1074* | ATGAACACGATTAACATCGCTAAGAACGACTTCTCTGACATCGAACTGGCTGCTATCCCGTTCAACACTCTGGCTGACCATTACGGTGAGCGTCTCGCTCGCGAACAGTTGGCCCTTGAGCATGAGTCTTACGAGATGGGTGAAGCACGCTTCCGCAAGATGTTTGAGCGTCAACTTAAAGCTGGTGAGGTTGCGGATAACGCTGCCGCCAAGCCTCTCATCACTACCCTACTCCCTAAGATGATTGCACGCATCAACGACTGGTTTGAGGAAGTGAAAGCTAAGCGCGGCAAGCGCCCGACAGCCTTCCAGTTCCTGCAAGAAATCAAGCCGGAAGCCGTAGCGTACATCACCATTAAGACCACTCTGGCTTGCCTAACCAGTGCTGACAATACAACCGTTCAGGCTGTAGCAAGCGCAATCGGTCGGGCCATTGAGGACGAGGCTCGCTTCGGTCGTATCCGTGACCTTGAAGCTAAGCACTTCAAGAAAAACGTTGAGGAACAACTCAACAAGCGCGTAGGGCACGTCTACAAGAAAGCATTTATGCAAGTTGTCGAGGCTGACATGCTCTCTAAGGGTCTACTCGGTGGCGAGGCGTGGTCTTCGTGGCATAAGGAAGACTCTATTCATGTAGGAGTACGCTGCATCGAGATGCTCATTGAGTCAACCGGAATGGTTAGCCTCCACCGCCAAAATGCTGGCGTAGTAGGTCAAGACTCTGAGACTATCGAACTCGCACCTGAATACGCTGAGGCTATCGCAACCCGTGCAGGTGCGCTGGCTGGCATCTCTCCGATGTTCCAACCTTGCGTAGTTCCTCCTAAGCCGTGGACTGGCATTACTGGTGGTGGCTATTGGGCTAACGGTCGTCGTCCTCTGGCGCTGGTGCGTACTCACAGTAAGAAAGCACTGATGCGCTACGAAGACGTTTACATGCCTGAGGTGTACAAAGCGATTAACATTGCGCAAAACACCGCATGGAAAATCAACAAGAAAGTCCTAGCGGTCGCCAACGTAATCACCAAGTGGAAGCATTGTCCGGTCGAGGACATCCCTGCGATTGAGCGTGAAGAACTCCCGATGAAACCGGAAGACATCGACATGAATCCTGAGGCTCTCACCGCGTGGAAACGTGCTGCCGCTGCTGTGTACCGCAAGGACAAGGCTCGCAAGTCTCGCCGTATCAGCCTTGAGTTCATGCTTGAGCAAGCCAATAAGTTTGCTAACCATAAGGCCATCTGGTTCCCTTACAACATGGACTGGCGCGGTCGTGTTTACGCTGTGTCAATGTTCAACCCGCAAGGTAACGATATGACCAAAGGACTGCTTACGCTGGCGAAAGGTAAACCAATCGGTAAGGAAGGTTACTACTGGCTGAAAATCCACGGTGCAAACTGTGCGGGTGTCGATAAGGTTCCGTTCCCTGAGCGCATCAAGTTCATTGAGGAAAACCACGAGAACATCATGGCTTGCGCTAAGTCTCCACTGGAGAACACTTGGTGGGCTGAGCAAGATTCTCCGTTCTGCTTCCTTGCGTTCTGCTTTGAGTACGCTGGGGTACAGCACCACGGCCTGAGCTATAACTGCTCCCTTCCGCTGGCGTTTGACGGGTCTTGCTCTGGCATCCAGCACTTCTCCGCGATGCTCCGAGATGAGGTAGGTGGTCGCGCGGTTAACTTGCTTCCTAGTGAAACCGTTCAGGACATCTACGGGATTGTTGCTAAGAAAGTCAACGAGATTCTACAAGCAGACGCAATCAATGGGACCGATAACGAAGTAGTTACCGTGACCGATGAGAACACTGGTGAAATCTCTGAGAAAGTCAAGCTGGGCACTAAGGCACTGGCTGGTCAATGGCTGGCTTACGGTGTTACTCGCAGTGTGACTAAGCGTTCAGTCATGACGCTGGCTTACGGGTCCAAAGAGTTCGGCTTCCGTCAACAAGTGCTGGAAGATACCATTCAGCCAGCTATTGATTCCGGCAAGGGTCTGATGTTCACTCAGCCGAATCAGGCTGCTGGATACATGGCTAAGCTGATTTGGGAATCTGTGAGCGTGACGGTGGTAGCTGCGGTTGAAGCAATGAACTGGCTTAAGTCTGCTGCTAAGCTGCTGGCTGCTGAGGTCAAAGATAAGAAGACTGGAGAGATTCTTCGCAAGCGTTGCGCTGTGCATTGGGTAACTCCTGATGGTTTCCCTGTGTGGCAGGAATACAAGAAGCCTATTCAGACGCGCTTGAACCTGATGTTCCTCGGTCAGTTCCGCCTCCAGCCTACCATTAACACCAACAAAGATAGCGAGATTGATGCACACAAACAGGAGTCTGGTATCGCTCCTAACTTTGTACACAGCCAAGACGGTAGCCACCTTCGTAAGACTGTAGTGTGGGCACACGAGAAGTACGGAATCGAATCTTTTGCACTGATTCACGACTCCTTCGGTACCATTCCGGCTGACGCTGCGAACCTGTTCAAAGCAGTGCGCGAAACTATGGTTGACACATATGAGTCTTGTGATGTACTGGCTGATTTCTACGACCAGTTCGCTGACCAGTTGCACGAGTCTCAATTGGACAAAATGCCAGCACTTCCGGCTAAAGGTAACTTGAACCTCCGTGACATCCTCGAGTCGGACTTCGCGTTCGCGTAA |

P_A6_：constitutive promoter drives transcription of RNAP in *C. glutamicum*

P_sal_：constitutive promoter drives transcription of RNAP in *E. coli*

Pre: constitutive promoter drives transcription of RNAP in *P. entemophila*

P_TAC_: inducible promoter drives transcription of RNAP or repressors in *C. glutamicum* and *E. coli*

P_lux_: inducible promoter drives transcription of RNAP in *P. entemophila*

P21-CuO: compositional promoter drives transcription of RNAP in *S. albus*

RBSs are indicated with lower-case letters.

**Supplementary Table 13. Sequences of crucial parts in the reporter plasmid backbones.**

| Ribozyme-based insulator | AGCTGTCACCGGATGTGCTTTCCGGTCTGATGAGTCCGTGAGGACGAAACAGCCTCTACAAATAATTTTGTTTAA |
| --- | --- |
| Composite terminator | CCAGGCATCAAATAAAACGAAAGGCTCAGTCGAAAGACTGGGCCTTTCGTTTTATCTGTTGTTTGTCGGTGAACGCTCTCTACTAGAGTCACACTGGCTCACCTTCGGGTGGGCCTTTCTGCGTTTATATACTAGAGCTGCTAACAAAGCCCGAAAGGAAGCTGAGTTGGCTGCTGCCACCGCTGAGCAATAACTAGCATAACCCCTTGGGGCCTCTAAACGGGTCTTGAGGGGTTTTTTGCTGAAAGGAGGAACTATATCCGGATTACTAGAGGTCATGCTTGCCATCTGTTTTCTTGCAAGAT |
| Reporter gene | tactagagaaagaggagaaatactagATGCGTAAAGGCGAAGAGCTGTTCACTGGTGTCGTCCCTATTCTGGTGGAACTGGATGGTGATGTCAACGGTCATAAGTTTTCCGTGCGTGGCGAGGGTGAAGGTGACGCAACTAATGGTAAACTGACGCTGAAGTTCATCTGTACTACTGGTAAACTGCCGGTACCTTGGCCGACTCTGGTAACGACGCTGACTTATGGTGTTCAGTGCTTTGCTCGTTATCCGGACCATATGAAGCAGCATGACTTCTTCAAGTCCGCCATGCCGGAAGGCTATGTGCAGGAACGCACGATTTCCTTTAAGGATGACGGCACGTACAAAACGCGTGCGGAAGTGAAATTTGAAGGCGATACCCTGGTAAACCGCATTGAGCTGAAAGGCATTGACTTTAAAGAAGACGGCAATATCCTGGGCCATAAGCTGGAATACAATTTTAACAGCCACAATGTTTACATCACCGCCGATAAACAAAAAAATGGCATTAAAGCGAATTTTAAAATTCGCCACAACGTGGAGGATGGCAGCGTGCAGCTGGCTGATCACTACCAGCAAAACACTCCAATCGGTGATGGTCCTGTTCTGCTGCCAGACAATCACTATCTGAGCACGCAAAGCGTTCTGTCTAAAGATCCGAACGAGAAACGCGATCATATGGTTCTGCTGGAGTTCGTAACCGCAGCGGGCATCACGCATGGTATGGATGAACTGTACAAATGA |
